# Quinone-catalyzed oxidative deformylation: synthesis of imines from amino alcohols

**DOI:** 10.3762/bjoc.13.282

**Published:** 2017-12-28

**Authors:** Xinyun Liu, Johnny H Phan, Benjamin J Haugeberg, Shrikant S Londhe, Michael D Clift

**Affiliations:** 1 Department of Chemistry, The University of Kansas, 2010 Malott Hall, 1251 Wescoe Hall Drive, Lawrence, KS, 66045, United States

**Keywords:** catalysis, deformylation, organic synthesis, organocatalysis

## Abstract

A new method for imine synthesis by way of quinone-catalyzed oxidative deformylation of 1,2-amino alcohols is reported. A wide range of readily accessible amino alcohols and primary amines can be reacted to provide N-protected imine products. The methodology presented provides a novel organocatalytic approach for imine synthesis and demonstrates the synthetic versatility of quinone-catalyzed oxidative C–C bond cleavage.

## Introduction

**Scheme 1:**
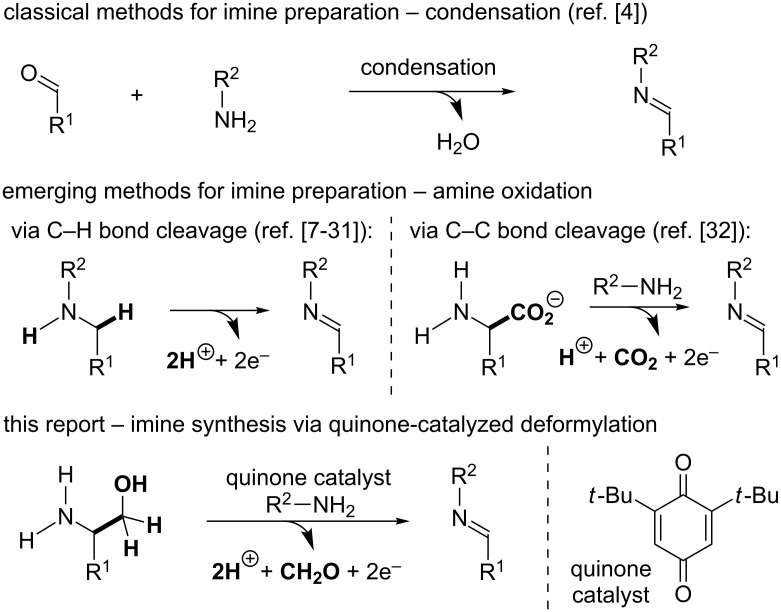
Established methods for the preparation of imines vs this work.

Imines are extremely versatile intermediates in organic chemistry [1–3]. Consequently, many synthetic methods have been developed for the preparation of imines (Scheme 1). The condensation of an amine with an aldehyde or ketone is the oldest and most commonly employed method for imine synthesis [4]. More recently, the catalytic dehydrogenation of amines mediated by metal and organic catalysts has begun to emerge as an alternative approach for the preparation of imines [5–6]. The majority of these methods involve cleavage of a C−H bond at the α-position of an amine substrate [7–28]. Methods that deliver imines through amine α-C−C bond cleavage are far less common [29–32] despite the fact that these methods employ renewable resources, such as amino acids and their derivatives, as starting materials. In fact, only a few reports describing the oxidative deformylation of amino alcohols have been published [33–35], and in all of these reports stoichiometric oxidants, such as NaIO_4_ and Pb(OAc)_4_, must be employed to enable the desired transformations. Given that 1,2-amino alcohols are readily accessible from feedstock chemicals such as styrenes [36–38] and amino acids [39], the development of a new methodology to transform these materials into high-value imine products under catalytic conditions has the potential to be broadly useful. Herein, we report a new method that utilizes quinone catalysis to enable the synthesis of imines via oxidative deformylation of amino alcohols.

Our group has recently reported the quinone-catalyzed decarboxylative homologation of α-amino acids [32], which demonstrated for the first time that quinone organocatalysts can be utilized to enable oxidative C–C bond cleavage to provide versatile imine intermediates. To further exploit the utility of this chemistry, we sought to develop a new method for the preparation of a wide range of imine products through the quinone-catalyzed deformylation of 1,2-amino alcohols. Such a transformation would not only facilitate rapid access to a variety of N-protected imines, but would also provide a novel approach for utilizing feedstock chemicals for the preparation of these valuable synthetic intermediates.

**Scheme 2:**
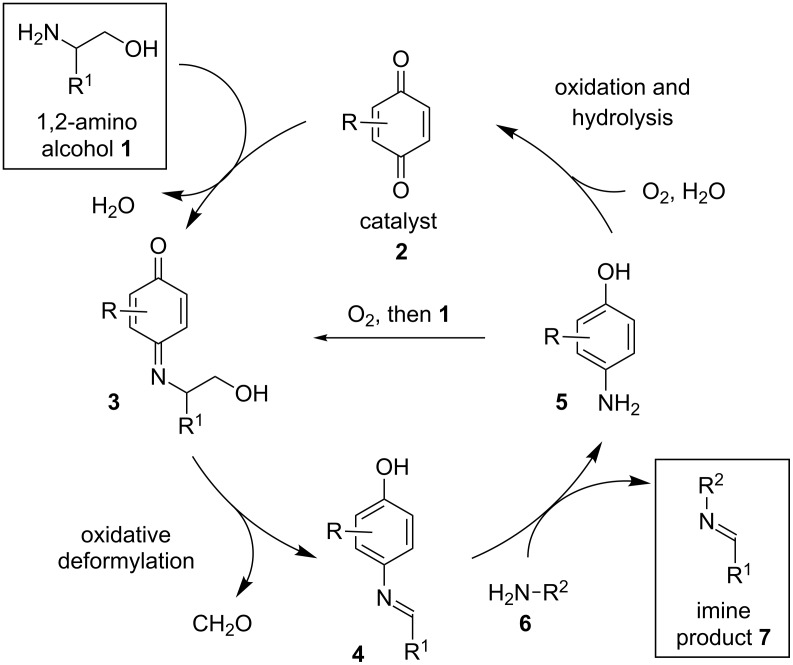
Proposed catalytic cycle for quinone-catalyzed deformylation.

We envisioned a process wherein a 1,2-amino alcohol **1** would undergo condensation with an appropriate quinone catalyst **2** to deliver iminoquinone **3** (Scheme 2). Deformylation of **3** would generate *N*-arylimine **4**. Subsequent transimination with amine **6** would provide the desired imine product **7** and a reduced form of the catalyst **5**, which would be expected to undergo oxidative turnover through one of two possible mechanisms (i.e., **5** → **3** or **5** → **2**).

## Results and Discussion

With this plan in mind, we first explored the ability of several quinone catalysts to promote the deformylation of 2-phenylglycinol (**1a**) to deliver *N*-PMP imine **7a** (Table 1). We selected quinone catalysts (**2a**−**c**) that have previously been utilized in amine oxidation reactions [21,32,40–41], and began with reaction conditions similar to those developed for our quinone-catalyzed oxidative decarboxylation chemistry [32]. To our delight, the desired deformylation product **7a** was formed in 63% yield when catalyst **2a** was employed (Table 1, entry 1). Quinone **2b** failed to deliver imine **7a** (Table 1, entry 2), but commercially available quinone **2c** provided **7a** in a promising 59% yield (Table 1, entry 3). Next, we examined the effect of base on the reaction using quinone **2c** as the catalyst (Table 1, entries 4−7). Unfortunately, no improvement in reaction efficiency was observed when different bases were employed (Table 1, entries 4−6, 0−55% yield); however, exclusion of the base provided imine **7a** in good yield (Table 1, entry 7, 85%). Decreasing the loading of catalyst **2c** under these conditions reduced the yield of imine **7a** (Table 1, entry 8, 64% yield), as did changing the identity of the catalyst (Table 1, entries 9 and 10, 62% and 0% respectively). Finally, we examined a range of solvents in an effort to further improve efficiency (Table 1, entries 11−17). No improvements in reaction efficiency were observed (Table 1, 0−72% yield), but it was noted that polar, protic solvents are critical in enabling the efficient deformylation of phenylglycinol.

**Table 1:** Optimization of quinone-catalyzed oxidative deformylation of phenylglycinol (**1a**).

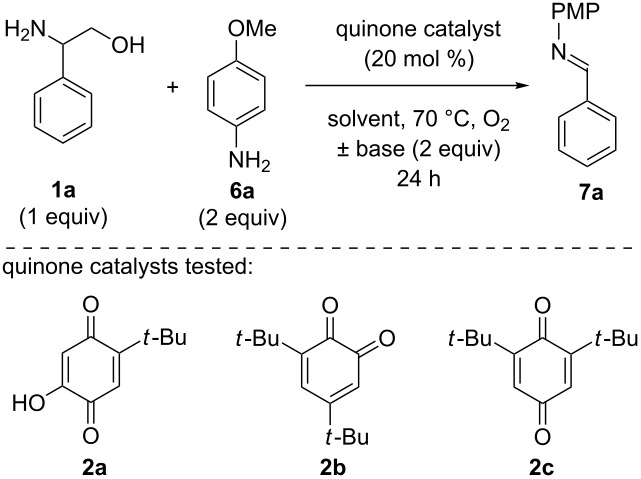				

Entry	Catalyst	Solvent	Base	Yield [%]^a^

1	**2a**	EtOH	Et_3_N	63
2	**2b**	EtOH	Et_3_N	0
3	**2c**	EtOH	Et_3_N	59
4	**2c**	EtOH	DABCO	55
5	**2c**	EtOH	DBU	0
6	**2c**	EtOH	K_2_CO_3_	17
7	**2c**	EtOH	none	85
8^b^	**2c**	EtOH	none	64
9	**2a**	EtOH	none	62
10	**2b**	EtOH	none	0
11	**2c**	iPrOH	none	72
12	**2c**	H_2_O	none	47
13	**2c**	MeCN	none	28
14	**2c**	DMSO	none	13
15^c^	**2c**	THF	none	0
16	**2c**	PhMe	none	11
17	**2c**	CHCl_3_	none	3

^a^Determined by ^1^H NMR using benzyl ether as an internal standard. ^b^10 mol % quinone was used. ^c^Reaction carried out at 50 °C.

With optimized conditions in hand, we next explored the scope of this methodology by employing a range of 1,2-amino alcohol substrates **1** (Table 2). As reported in Table 1, the reaction involving phenylglycinol gave the desired *N*-PMP imine (**7a**) in 85% yield (Table 2, entry 1). *ortho*-Substitution of the arene is reasonably well-tolerated, as 2-methylphenylglycinol (**1b**) and 2-chlorophenylglycinol (**1c**) delivered the corresponding imines in 68% yield (Table 2, entries 2 and 3). The *meta*-fluoro derivative provided imine **7d** in 60% yield (Table 2, entry 4). Electronic effects were studied by examining a series of *para*-substituted phenylglycinol derivatives (Table 2, entries 5−9). Both electron-donating and electron-withdrawing substituents were tolerated, but no obvious trends in the reactivity patterns were observed (47−77% yield). Thiophenyl amino alcohol **1j** was also subjected to the optimized conditions and the corresponding imine **7j** was formed in 47% yield (Table 2, entry 10). Unfortunately, aliphatic 1,2-amino alcohols, such as valinol (**1k**), failed to undergo deformylation under the current conditions (Table 2, entry 11).

**Table 2:** Quinone-catalyzed oxidative deformylation of various amino alcohols.

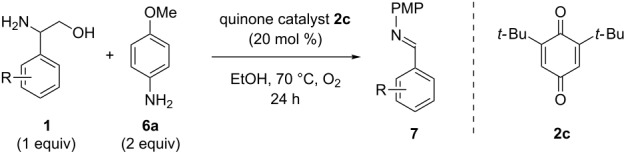					

Entry	Amino alcohol **1**		Product **7**		Yield [%]^a^

1 2 3	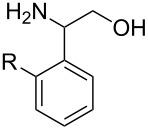	**1a**, R = H **1b**, R = Me **1c**, R = Cl	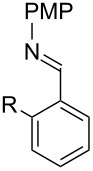	**7a**, R = H **7b**, R = Me **7c**, R = Cl	85 68 68
4	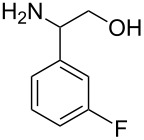	**1d**	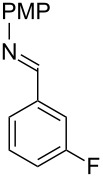	**7d**	60
5 6 7 8 9	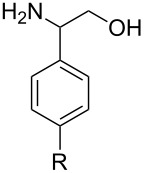	**1e**, R = Me **1f**, R = OMe **1g**, R = Cl **1h**, R = F **1i**, R = CF_3_	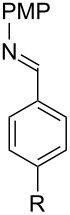	**7e**, R = Me **7f**, R = OMe **7g**, R = Cl **7h**, R = F **7i**, R = CF_3_	68 77 66 54 47
10	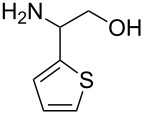	**1j**	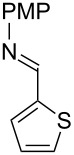	**7j**	47
11	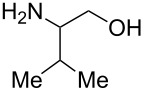	**1k**	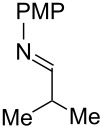	**7k**	0

^a^Determined by ^1^H NMR using benzyl ether as an internal standard (average of two replicates).

Next, we investigated the use of various amine reaction partners **6** to access a variety of imine products **7** from phenylglycinol (**1a**, Table 3). The reaction with aniline (**6l**, Table 3, entry 2, 68% yield) showed reduced reaction efficiency compared to that with *para*-anisidine (**6a**, Table 3, entry 1, 85% yield). When *para*-fluoroaniline (**6m**) was employed as the reaction partner, imine **7m** was produced in a 77% yield (Table 3, entry 3). α-Branched amines are effective reaction partners, providing the corresponding imines **7n**−**p** in modest yields (Table 3, entries 4–6, 42−66% yield). From these results, it can be concluded that increasing the steric bulk at the α-position of the amine results in decreased reaction efficiency. Phenethylamine (**6q**) provided only a 17% yield of the corresponding imine (**7q**, Table 3, entry 7), potentially due to its increased nucleophilicity, which may result in inhibition of catalysis via condensation with quinone **2c**. We also tested several electron deficient amides (**6r**−**t**) in these reactions (Table 3, entries 8−10). Unfortunately, only sulfinamide **6t** provided the desired imine **7t** (Table 3, entry 10, 22% yield). In all three cases, a significant amount of benzaldehyde was observed, indicating that electron deficient primary amides (such as **6r**−**t**) are either incapable of promoting transimination, or the resulting imines (**7r**−**t**) are hydrolyzed under the current reaction conditions. Notably, imines **7n** [42–45] and **7t** [46–48] are useful imines for diastereoselective 1,2-addition reactions.

**Table 3:** Quinone-catalyzed oxidative deformylation using various amine reaction partners.

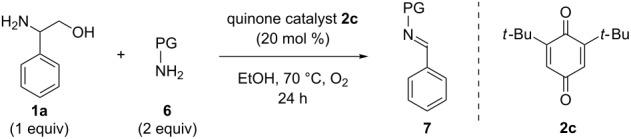					

Entry	Amine **6**		Product **7**		Yield [%]^a^

1 2 3	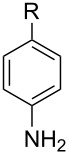	**6a**, R = OMe **6l**, R = H **6m**, R = F	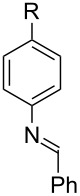	**7a**, R = OMe **7l**, R = H **7m**, R = F	85 68 77
4	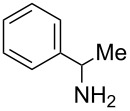	**6n**	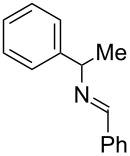	**7n**	66
5	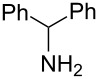	**6o**	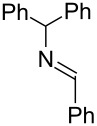	**7o**	42
6	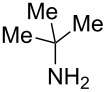	**6p**	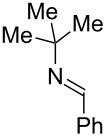	**7p**	56
7	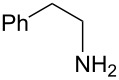	**6q**	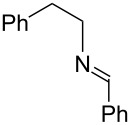	**7q**	17
8	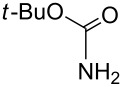	**6r**	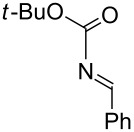	**7r**	0
9	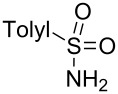	**6s**	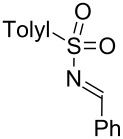	**7s**	0
10	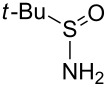	**6t**	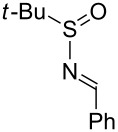	**7t**	22

^a^Determined by ^1^H NMR using benzyl ether as an internal standard (average of two replicates).

**Scheme 3:**
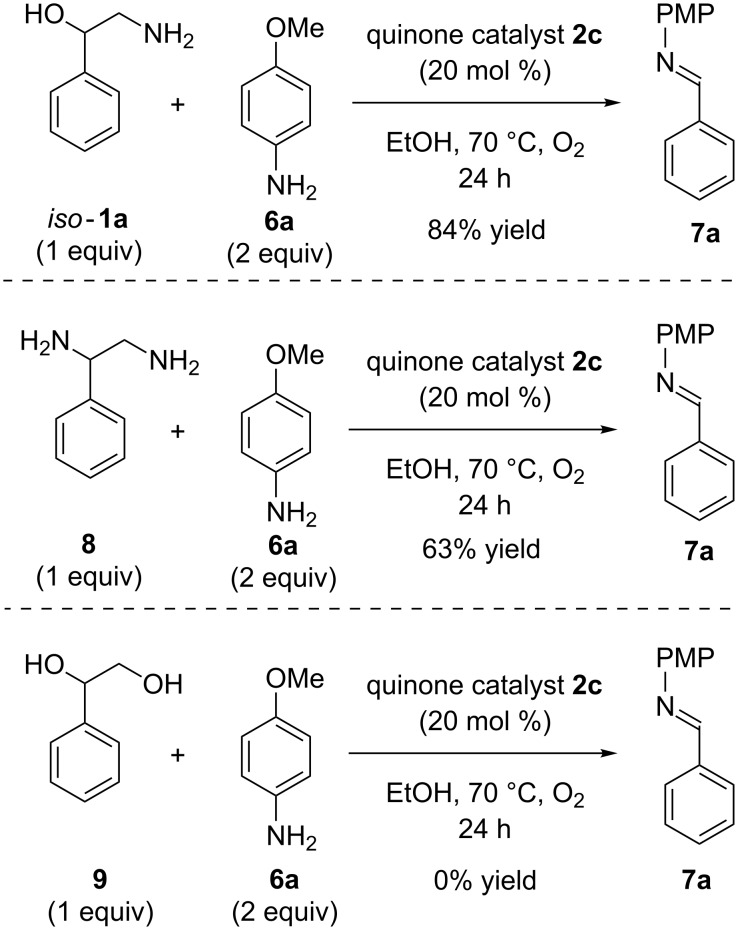
Studies of quinone-catalyzed C−C bond cleavage in related substrates.

Following these substrate scope studies, we next examined the quinone-catalyzed C–C bond cleavage of analogous substrates (Scheme 3). First, we tested isomeric amino alcohol *iso-***1a**, which provided imine **7a** in a yield comparable to that observed when phenylglycinol was used as a substrate. Notably, the mechanism of this reaction likely involves initial formation of benzaldehyde, followed by condensation with *para*-anisidine, to deliver imine **7a**. Vicinal diamine **8** was also a compatible substrate, delivering imine **7a** in 63% yield. Finally, we subjected diol **9** to the optimal reaction conditions; no product was observed, indicating that condensation between the substrate and catalyst to form an iminoquinone intermediate is likely required for productive reactivity.

**Scheme 4:**
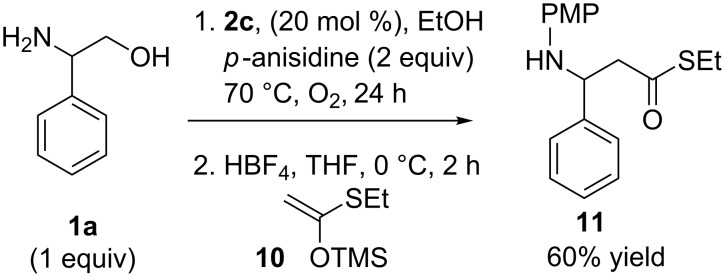
Sequential oxidative deformylation/Mukaiyama−Mannich addition using phenylglycinol.

To demonstrate the synthetic utility of this methodology, we performed a sequential oxidative deformylation/Mukaiyama−Mannich addition under our previously reported conditions for decarboxylative amino acid homologation (Scheme 4) [32]. In this reaction sequence, (thio)silyl ketene acetal **10** was united with 2-phenylglycinol and *para*-anisidine in a two-step, one-pot process to provide β-amino acid derivative **11** in a 60% yield. The overall reaction sequence provides a unique method for the production of the high-value β-amino acid derivatives [49–50] from 1,2-amino alcohols.

## Conclusion

In conclusion, we have developed a novel method for the synthesis of imines from 1,2-amino alcohols. This chemistry features an unprecedented application of quinone organocatalysis to enable oxidative deformylation under aerobic conditions. Future work will involve mechanistic studies and the development of new catalysts to expand the scope of this chemistry.

## Supporting Information

File 1 Experimental procedures, compound characterization data, and copies of ^1^H and ^13^C NMR spectra.
