# Supplementary material for: Quinone-catalyzed oxidative deformylation: synthesis of imines from amino alcohols
Source: Beilstein J Org Chem. 2017 Dec 28;13:2895–901. doi: 10.3762/bjoc.13.282 (PMC5753116; doi:10.3762/bjoc.13.282)
Supplement: File 1 — Experimental procedures, compound characterization data, and copies of 1H and 13C NMR spectra. [file Beilstein_J_Org_Chem-13-2895-s001.pdf]

Supporting Information

for

Quinone-catalyzed oxidative deformylation: synthesis of imines from amino alcohols

Xinyun Liu, Johnny H. Phan, Benjamin J. Haugeberg, Shrikant S. Londhe and Michael D. Clift*[1]

Address: Department of Chemistry, The University of Kansas, 2010 Malott Hall, 1251 Wescoe Hall Drive, Lawrence, KS, 66045, United States

Email: Michael D. Clift - mclift@ku.edu

*Corresponding author

Experimental procedures, compound characterization data, and copies of ^{1}H and ^{13}C NMR spectra

1. General methods S1–S2
2. General experimental procedures for oxidative deformylation S2–S2
3. Experimental procedures and characterization data for **7a−j, 7l−q, 7t** and **11** S3–S8
4. ^{1}H and ^{13}C NMR spectra S9–S26

Part 1: General methods

All reactions were carried out in 10 mL round bottom flasks or 8 mL reaction vials with magnetic stirring. EtOH was used as purchased from Fisher Chemical (190 Proof). Other reagents were used as delivered from commercial sources. Purification of reaction products was carried out by flash chromatography using Fisher Chemical silica gel (230–400 Mesh, Grade 60). Analytical thin-layer chromatography (TLC) was performed on EMD millipore TLC silica gel 60 – F254: 25 glass plates. Visualization was accomplished with UV light and/or phosphomolybdic acid staining followed by heating. Melting point

data was recorded using a Digimelt SRS. Film infrared (IR) spectra were recorded using a Shimadzu FTIR-8400S. ^{1}H NMR spectra were recorded on Bruker 500 (500 MHz) or Bruker 600 (600 MHz) spectrometers and are reported in ppm using solvent as a reference ($CDCl_3$ at 7.26 ppm). Proton-decoupled ^{13}C NMR spectra were recorded on a Bruker 500 (125 MHz) or Bruker 600 (150 MHz) spectrometers and are reported in ppm using solvent as a reference ($CDCl_3$ at 77.16 ppm). NMR data are reported as chemical shift (app = apparent, s = singlet, d = doublet, t = triplet, q = quartet, m = multiplet, br = broad), integration, and coupling constant(s) in Hz. Mass spectra data were obtained on a Micromass Ltd. LCT Premier quadrupole and time-of-flight tandem mass analyzer.

Part 2: General experimental procedures for oxidative deformylation

A: General procedure for the synthesis of imines 7a−k: To a solution of 2,6-di-*tert*-butyl-1,4-benzoquinone (**2c**, 0.20 mmol, 44 mg, 0.2 equiv) and *p*-anisidine (**6a**, 2.0 mmol, 246 mg, 2.0 equiv) in ethanol (3.2 mL) was added the 1,2-amino alcohol **1** (1.0 mmol). The flask was purged with O_2 and then heated to 70 °C with vigorous stirring for 24 h. After the reaction mixture was cooled to room temperature, benzyl ether (1.0 mmol, 190 µL, 1.0 equiv) was added as an internal standard and the reaction was concentrated under reduced pressure. ^{1}H NMR was taken in $CDCl_3$ and the spectrum obtained was used to assess the yield of imine **7**. The desired imine product was subsequently purified by flash chromatography on triethylamine treated silica gel (eluent: 10% ethyl acetate in hexanes) to enable characterization of the imine product **7** through ^{1}H NMR, ^{13}C NMR, IR and MS.

B: General procedure for the synthesis of imines 7l−t: To a solution of 2,6-di-*tert*-butyl-1,4-benzoquinone (**2c**, 0.10 mmol, 22 mg, 0.2 equiv) and amine **6** (1.0 mmol, 2.0 equiv) in ethanol (1.6 mL) was added phenylglycinol (**1a**, 0.50 mmol, 68.6 mg). The vial was purged with O_2 and then heated to 70 °C with vigorous stirring for 24 h. After the reaction mixture was cooled to room temperature, benzyl ether (0.50 mmol, 95 µL, 1.0 equiv) was added as an internal standard and the reaction was concentrated under reduced pressure. ^{1}H NMR was taken in $CDCl_3$ and the spectrum was used to assess the yield of imine (**7l−t**). The desired imine product was subsequently purified by flash chromatography on triethylamine treated silica gel (eluent: 10% ethyl acetate in hexanes) to enable characterization of the imine product **7** through ^{1}H NMR, ^{13}C NMR, IR and MS.

C: General procedure for oxidative C–C bond cleavage of related substrates (*iso*-1a, 8−9): To a solution of 2,6-di-*tert*-butyl-1,4-benzoquinone (**2c**, 0.10 mmol, 22 mg, 0.2 equiv) and *p*-anisidine (**6a**, 1.0 mmol, 123 mg, 2.0 equiv) in ethanol (1.6 mL) was added the substrate (*iso*-**1a**, **8**, or **9**, 0.50 mmol). The vial was purged with O_2 and then heated to 70 °C with vigorous stirring for 24 h. After the reaction mixture was cooled to room temperature, benzyl ether (0.50 mmol, 95 µL, 1.0 equiv) was added as an internal standard and the reaction was concentrated under reduced pressure. ^{1}H NMR was taken in $CDCl_3$ and the spectrum was used to assess the yield of imine (**7a**).

Part 3: Experimental procedures and characterization data for 7a−j, 7l-q, 7t and 11

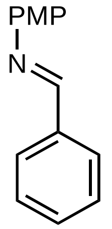

(*E*)-*N*-(4-Methoxyphenyl)-1-phenylmethanimine (7a): The reaction was carried out according to the general procedure **(A)** using 2-phenylglycinol (137.2 mg, 1.0 mmol). A comparison of the ^{1}H NMR integrals for the imine proton of **7a** and the benzylic protons of benzyl ether indicated that **7a** was obtained in 85% yield in trial #1, and 85% yield in trial #2, for an average yield of 85%. Following purification, product **7a** was isolated as an orange solid, m.p. 70 °C. IR (film) 3024, 2953, 1622, 1207 cm^{-1}. ^{1}H NMR (500 MHz, $CDCl_3$) 8.49 (s, 1H), 7.90 (m, 2H), 7.50 – 7.42 (m, 3H), 7.27 – 7.25 (m, Ar H, obscured by $CDCl_3$), 7.01 – 6.89 (m, 2H), 3.84 (s, 3H). ^{13}C NMR (126 MHz, $CDCl_3$) 158.5, 158.3, 144.9, 136.4, 131.1, 128.8, 128.6, 122.2, 114.4, 55.5; HRMS (ESI): Exact mass calcd for $C_{14}H_{14}NO$ [M+H$^+$], 212.1075. Found 212.1056. This data is consistent with the data previously reported for compound **7a.**[1]

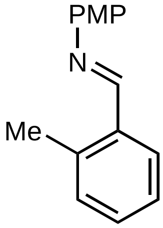

(*E*)-*N*-(4-Methoxyphenyl)-1-(*o*-tolyl)methanimine (7b): The reaction was carried out according to the general procedure (**A**) using 2-amino-2-(o-tolyl)ethan-1-ol[2] (151.2 mg, 1.0 mmol). A comparison of the ^{1}H NMR integrals for the imine proton of **7b** and the benzylic protons of benzyl ether indicated that **7b** was obtained in 69% yield in trial #1, and 67% yield in trial #2, for an average yield of 68%. Following purification, product **7b** was isolated as an orange solid, m.p. 56 °C; IR (film) 3030, 2991, 1618, 1209 cm^{-1}. ^{1}H NMR (500 MHz, $CDCl_3$) 8.77 (s, 1H), 8.07 (dd, J = 7.6, 1.6 Hz, 1H), 7.35 (app td, 1H), 7.29 (app td, 1H), 7.24 – 7.20 (m, 3H), 6.97 – 6.91 (m, 2H), 3.84 (s, 3H), 2.59 (s, 3H). ^{13}C NMR (126 MHz, $CDCl_3$) 158.3, 157.3, 145.7, 138.5, 134.5, 131.1, 130.9, 127.6, 126.5, 122.3, 114.5, 55.7, 19.6; HRMS (ESI): Exact mass calcd for $C_{15}H_{16}NO$ [M+H$^+$], 226.1232. Found 226.1211. This data is consistent with the data previously reported for compound **7b.**[3]

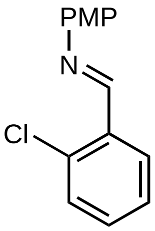

(*E*)-1-(2-Chlorophenyl)-*N*-(4-methoxyphenyl)methanimine (7c): The reaction was carried out according to the general procedure (**A**) using 2-amino-2-(2-chlorophenyl)ethan-1-ol[2] (171.6 mg, 1.0 mmol). A comparison of the ^{1}H NMR integrals for the imine proton of **7c** and the benzylic protons of benzyl ether indicated that **7c** was obtained in 67% yield in trial #1, and 68% yield in trial #2, for an average yield of 68%. Following purification, product **7c** was isolated as an orange solid, m.p. 61 °C. IR (film) 3059, 2949, 1616, 1207 cm^{-1}. ^{1}H NMR (500 MHz, $CDCl_3$) 8.94 (s, 1H), 8.29 – 8.15 (m, 1H), 7.44 – 7.41 (m, 1H), 7.40 – 7.34 (m, 2H), 7.30 – 7.26 (m, 2H), 6.97 – 6.92 (m, 2H), 3.85 (s, 3H). ^{13}C NMR (126 MHz, $CDCl_3$) 158.8, 154.9, 144.8, 136.0, 133.6, 131.9, 130.1, 128.5, 127.2, 122.7, 114.6, 55.7; HRMS (ESI): Exact mass calcd for $C_{14}H_{13}NOCl$ [M+H$^+$], 246.0686. Found 246.0666. This data is consistent with the data previously reported for compound **7c**.[4]

---

[1] Keinicke, L.; Fristrup, P.; Norrby, P.; Madsen, R.; *J. Am. Chem. Soc.* **2005,** *127*, 15756-15761.

[2] Prepared according to: Feng, D.; Song, Y.; Jiang, X.; Chen, L.; Long, Y. *Org. Biomol. Chem.* **2007**, *5*, 2690−2697.

[3] Mu, D; Wang, X.; Chen, G.; He, G.; *J. Org. Chem.* **2017,** *82,* 4497-4503.

[4] Divse, J. M.; Mhaske, S. B.; Charolkar, C. R.; Sant, D. T.; Deshpande, M. V.; Khedkar, V. M.; Nawale, L. U.; Sarkar, D.; Pore, V. S.; *New J. Chem.* **2017,** *41,* 470-479.

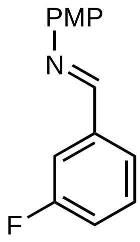

(*E*)-1-(3-Fluorophenyl)-*N*-(4-methoxyphenyl)methanimine (7d): The reaction was carried out according to the general procedure (**A**) using 2-amino-2-(3-fluorophenyl)ethan-1-ol[2] (155.2 mg, 1.0 mmol). A comparison of the ^{1}H NMR integrals for the imine proton of **7d** and the benzylic protons of benzyl ether indicated that **7d** was obtained in 61% yield in trial #1, and 59% yield in trial #2, for an average yield of 60%. Following purification, product **7d** was isolated as an orange solid, m.p. 65 ^{o}C. IR (film) 3018, 2960, 1622, 1205 cm^{-1}. ^{1}H NMR (500 MHz, $CDCl_3$) 8.46 (d, J = 1.3 Hz, 1H), 7.65 (ddd, J = 9.5, 2.7, 1.4 Hz, 1H), 7.61 (app dt, 1H), 7.43 (app td, 1H), 7.26 – 7.24 (m, Ar H, obscured by $CDCl_3$), 7.16 (app tdd, 1H), 6.96 – 6.92 (m, 2H), 3.84 (s, 3H). ^{13}C NMR (126 MHz, $CDCl_3$) 163.2 (d, J = 246.6 Hz), 158.7, 156.8 (d, J = 3.1 Hz), 144.4, 138.9 (d, J = 7.5 Hz), 130.4 (d, J = 8.1 Hz), 124.9 (d, J = 2.7 Hz), 122.4, 118.1 (d, J = 21.8 Hz), 114.56 (d, J = 22.4 Hz), 114.55, 55.7 ; HRMS (ESI): Exact mass calcd for $C_{14}H_{13}NFO$ [M+H$^+$], 230.0981. Found 230.0958. This data is consistent with the data previously reported for compound **7d**.[5]

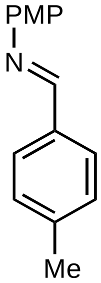

(*E*)-*N*-(4-Methoxyphenyl)-1-(*p*-tolyl)methanimine (7e): The reaction was carried out according to the general procedure (**A**) using 2-amino-2-(p-tolyl)ethan-1-ol[2] (151.2 mg, 1.0 mmol). A comparison of the ^{1}H NMR integrals for the imine proton of **7e** and the benzylic protons of benzyl ether indicated that **7e** was obtained in 68% yield in trial #1, and 68% yield in trial #2, for an average yield of 68%. Following purification, product **7e** was isolated as an orange solid, m.p. 85 ^{o}C. IR (film) 3010, 2914, 1624, 1209 cm^{-1}. ^{1}H NMR (500 MHz, $CDCl_3$) 8.44 (s, 1H), 7.78 (d, J = 8.1 Hz, 2H), 7.27 (d, Ar H, obscured by $CDCl_3$), 7.24 – 7.21 (m, 2H), 6.95 – 6.91 (m, 2H), 3.83 (s, 3H), 2.41 (s, 3H).^{13}C NMR (126 MHz, $CDCl_3$) 158.6, 158.2, 145.2, 141.6, 134.0, 129.6, 128.7, 122.3, 114.5, 55.6, 21.8; HRMS (ESI): Exact mass calcd for $C_{15}H_{16}NO$ [M+H$^+$], 226.1232. Found 226.1205. This data is consistent with the data previously reported for compound **7e**.[5]

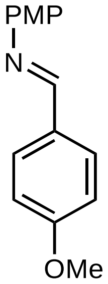

(*E*)-*N*,1-bis(4-Methoxyphenyl)methanimine (7f): The reaction was carried out according to the general procedure (**A**) using 2-amino-2-(4-methoxyphenyl)ethan-1-ol[2] (167.2 mg, 1.0 mmol). A comparison of the ^{1}H NMR integrals for the imine proton of **7f** and the benzylic protons of benzyl ether indicated that **7f** was obtained in 78% yield in trial #1, and 76% yield in trial #2, for an average yield of 77%. Following purification, product **7f** was isolated provide after purification product **7f** as a pale yellow solid, m.p. 145 ^{o}C. IR (film) 3012, 2953, 1622, 1209 cm^{-1}. ^{1}H NMR (500 MHz, $CDCl_3$) 8.41 (s, 1H), 7.83 (d, J = 8.8 Hz, 2H), 7.21 (d, J = 8.9 Hz, 2H), 6.98 (d, J = 8.8 Hz, 2H), 6.92 (d, J = 9.0 Hz, 2H), 3.87 (s, 3H), 3.83 (s, 3H). ^{13}C NMR (126 MHz, $CDCl_3$) 162.1, 158.1, 158.0, 145.4, 130.3, 129.6, 122.2, 114.5, 114.3, 55.64, 55.56; HRMS (ESI): Exact mass calcd for $C_{15}H_{16}NO_2$ [M+H$^+$], 242.1181. Found 242.1156. This data is consistent with the data previously reported for compound **7f**.[5]

[5] Hong, X.; Wang, H.; Liu, B.; Xu, B.; *Chem. Commun.* **2014,** *50,* 14129-14132.

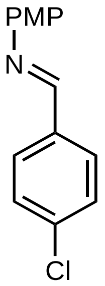

(*E*)-1-(4-Chlorophenyl)-*N*-(4-methoxyphenyl)methanimine (7g): The reaction was carried out according to the general procedure (**A**) using 2-amino-2-(4-chlorophenyl)ethan-1-ol[2] (171.6 mg, 1.0 mmol). A comparison of the ^{1}H NMR integrals for the imine proton of **7g** and the benzylic protons of benzyl ether indicated that **7g** was obtained in 62% yield in trial #1, and 70% yield in trial #2, for an average yield of 66%. Following purification, product **7g** was isolated as an orange solid, m.p. 123 ^{o}C. IR (film) 3032, 2956, 1620, 1207 cm^{-1}. ^{1}H NMR (500 MHz, $CDCl_3$) 8.45 (s, 1H), 7.83 (d, J = 8.5 Hz, 2H), 7.44 (d, J = 8.5 Hz, 2H), 7.25 – 7.22 (m, 2H), 6.96 – 6.92 (m, 2H), 3.84 (s, 3H). ^{13}C NMR (126 MHz, $CDCl_3$) 158.6, 156.9, 144.6, 137.1, 135.1, 129.8, 129.2, 122.4, 114.5, 55.7; HRMS (ESI): Exact mass calcd for $C_{14}H_{13}NOCl$ [M+H^{+}], 246.0686. Found 246.0668. This data is consistent with the data previously reported for compound **7g**.[5]

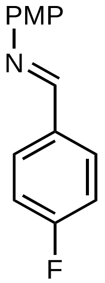

(*E*)-1-(4-Fluorophenyl)-*N*-(4-methoxyphenyl)methanimine (7h): The reaction was carried out according to the general procedure (**A**) using 2-amino-2-(4-fluorophenyl)ethan-1-ol[2] (155.2 mg, 1.0 mmol). A comparison of the ^{1}H NMR integrals for the imine proton of **7h** and the benzylic protons of benzyl ether indicated that **7h** was obtained in 55% yield in trial #1, and 53% yield in trial #2, for an average yield of 54%. Following purification, product **7h** was isolated as an orange solid, m.p. 96 ^{o}C. IR (film) 3014, 2964, 1624, 1209 cm^{-1}. ^{1}H NMR (500 MHz, $CDCl_3$) 8.45 (s, 1H), 7.93 – 7.83 (m, 2H), 7.25 – 7.20 (m, 2H), 7.18 – 7.12 (m, 2H), 6.98 – 6.88 (m, 2H), 3.84 (s, 3H). ^{13}C NMR (126 MHz, $CDCl_3$) 164.6 (d, J = 251.6 Hz), 158.4, 157.0, 144.8, 132.9 (d, J = 3.0 Hz), 130.6 (d, J = 8.7 Hz), 122.3, 116.0 (d, J = 21.9 Hz), 114.5, 55.6; HRMS (ESI): Exact mass calcd for $C_{14}H_{13}NOF$ [M+H^{+}], 230.0981. Found 230.0958. This data is consistent with the data previously reported for compound **7h**.[5]

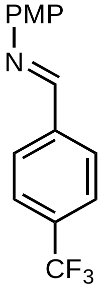

(*E*)-*N*-(4-Methoxyphenyl)-1-(4-(trifluoromethyl)phenyl)methanimine (7i): The reaction was carried out according to the general procedure (**A**) using 2-amino-2-(4-(trifluoromethyl)phenyl)ethan-1-ol[2] (205.2 mg, 1.0 mmol). A comparison of the ^{1}H NMR integrals for the imine proton of **7i** and the benzylic protons of benzyl ether indicated that **7i** was obtained in 45% yield in trial #1, and 48% yield in trial #2, for an average yield of 47%. Following purification, product **7i** was isolated as an orange solid, m.p. 121 ^{o}C. IR (flim) 3064, 2943, 1614, 1211 cm^{-1}. ^{1}H NMR (500 MHz, $CDCl_3$) 8.54 (s, 1H), 8.00 (d, *J* = 8.0 Hz, 2H), 7.72 (d, *J* = 8.0 Hz, 2H), 7.26 (d,Ar H, obscured by $CDCl_3$), 6.95 (d, *J* = 8.4 Hz, 2H), 3.85 (s, 3H).^{13}C NMR (126 MHz, $CDCl_3$) 158.9, 156.4, 144.2, 139.6, 132.5 (app d), 130.1, 128.84 125.82 (q, J = 3.8 Hz), 122.5, 114.6, 55.7; HRMS (ESI): Exact mass calcd for $C_{15}H_{13}NOF_3$ [M+H^{+}], 280.0949. Found 280.0919. This data is consistent with the data previously reported for compound **7i**.[6]

[6] Lai, J.; Yang, Y.; Lin, J.; Yang, D.; *Synlett* **2010,** *1,* 111-114.

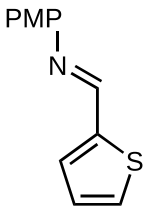

(*E*)-*N*-(4-Methoxyphenyl)-1-(thiophen-2-yl)methanimine (7j): The reaction was carried out according to the general procedure (**A**) using 2-amino-2-(thiophen-2-yl)ethan-1-ol[2] (143.2 mg, 1.0 mmol). A comparison of the ^{1}H NMR integrals for the imine proton of **7j** and the benzylic protons of benzyl ether indicated that **7j** was obtained in 44% yield in trial #1, and 49% yield in trial #2, for an average yield of 47%. Following purification, product **7j** was isolated as a brown solid, m.p. 45 ^{o}C. IR (film) 3032, 2962, 1622, 1209 cm^{-1}. ^{1}H NMR (500 MHz, $CDCl_3$) 8.54 (s, 1H), 8.12 – 7.99 (m, 2H), 7.72 (d, J = 8.0 Hz, 2H), 7.29 – 7.26 (m, Ar H, obscured by $CDCl_3$), 7.16 – 6.79 (m, 2H), 3.85 (s, 3H). ^{13}C NMR (126 MHz, $CDCl_3$) 158.9, 156.4, 144.2, 139.7, 128.8, 125.84, 125.81, 122.5, 114.6, 55.7; HRMS (ESI): Exact mass calcd for $C_{12}H_{12}NOS$ [M+H^{+}], 218.0640. Found 218.0632.

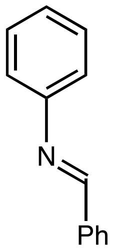

(*E*)-*N*,1-Diphenylmethanimine (7l): The reaction was carried out according to the general procedure (**B**) using aniline (91 µL, 1.0 mmol). A comparison of the ^{1}H NMR integrals for the imine proton of **7l** and the benzylic protons of benzyl ether indicated that **7l** was obtained in 71% yield in trial #1, and 65% yield in trial #2, for an average yield of 68%. Following purification, product **7l** was isolated as a pale yellow solid, m.p. 50 ^{o}C. IR (film) 3294, 1629 cm^{-1}. ^{1}H NMR (500 MHz, $CDCl_3$) 8.47 (s, 1H), 7.99 – 7.82 (m, 2H), 7.49 (dd, *J* = 5.2, 2.0 Hz, 3H), 7.41 (m, 2H), 7.25 – 7.20 (m, Ar H, obscured by $CDCl_3$).^{13}C NMR (125 MHz, $CDCl_3$) 160.5, 131.6, 129.3, 129.0, 128.9, 126.1, 121.0; HRMS (ESI): Exact mass calcd for $C_{13}H_{12}N$ [M+H^{+}], 182.0970. Found 182.0957. This data is consistent with the data previously reported for compound **7l**.[5]

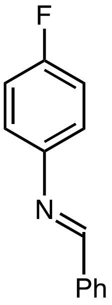

(*E*)-*N*-(4-Fluorophenyl)-1-phenylmethanimine (7m): The reaction was carried out according to the general procedure (**B**) using 4-fluoroaniline (95 µL, 1.0 mmol). A comparison of the ^{1}H NMR integrals for the imine proton of **7m** and the benzylic protons of benzyl ether indicated that **7m** was obtained in 77% yield in trial #1, and 77% yield in trial #2, for an average yield of 77%. Following purification, product **7m** was isolated as a pale yellow solid, m.p. 57 ^{o}C. IR (film) 3039, 1645 cm^{-1}. ^{1}H NMR (500 MHz, $CDCl_3$) 8.45 (s, 1H), 7.98 – 7.81 (m, 2H), 7.49 (m, 3H), 7.24 – 7.19 (m, 2H), 7.13 – 7.05 (m, 2H). ^{13}C NMR (125 MHz, $CDCl_3$) 161.3 (d, *J* = 244.5 Hz), 160.1, 135.2 (d, *J* = 214.3 Hz), 131.4, 129.7, 129.0, 128.8, 122.3 (d, *J* = 8.2 Hz), 115.8 (d, *J* = 22.6 Hz); HRMS (ESI): Exact mass calcd for $C_{13}H_{11}NF$ [M+H^{+}], 200.0876. Found 200.0844. This data is consistent with the data previously reported for compound **7m**.[7]

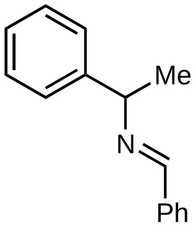

(*E*)-1-Phenyl-*N*-(1-phenylethyl)methanimine (7n): The reaction was carried out according to the general procedure (**B**) using 1-phenylethan-1-amine (129 µL, 1.0 mmol). A comparison of the ^{1}H NMR integrals for the imine proton of **7n** and the benzylic protons of benzyl ether indicated that **7n** was obtained in 62% yield in trial #1, and 70% yield in trial #2, for an average yield of 66%. Following purification, product **7n** was isolated as a colorless liquid. IR (film) 3058, 2968, 1643 cm^{-1}. ^{1}H NMR (600 MHz, $CDCl_3$) 8.38 (s, 1H), 7.79 (dd, *J* = 6.7, 3.0 Hz, 2H), 7.44 (d, *J* = 8.0, 2H), 7.41 (m, 3H), 7.35 (app t, 2H), 7.24 (Ar H, obscured by $CDCl_3$),

---

[7] Sadownik, J. W.; Philp, D.; *Angew. Chem. Int. Ed.* **2008,** *47,* 9965-9970.

4.56 (q, *J* = 6.7 Hz, 1H), 1.60 (d, *J* = 6.7 Hz, 3H). ^{13}C NMR (150 MHz, $CDCl_3$) 159.6, 145.3, 136.6, 130.7, 128.7, 128.5, 128.4, 127.0, 126.8, 69.9, 25.0; HRMS (ESI): Exact mass calcd for $C_{15}H_{16}N$ [M+H$^+$], 210.1283. Found 210.1274. This data is consistent with the data previously reported for compound **7n**.[8]

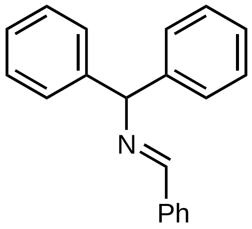

(*E*)-*N*-Benzhydryl-1-phenylmethanimine (7o): The reaction was carried out according to the general procedure (**B**) using diphenylmethanamine (172 µL, 1.0 mmol). A comparison of the ^{1}H NMR integrals for the imine proton of **7o** and the benzylic protons of benzyl ether indicated that **7o** was obtained in 42% yield in trial #1, and 42% yield in trial #2, for an average yield of 42%. Following purification, product **7o** was isolated as a white solid, m.p. 95 ^{o}C. IR (film) 3026, 2866, 1637 cm^{-1}. ^{1}H NMR (600 MHz, $CDCl_3$) 8.44 (s, 1H), 7.90 – 7.79 (m, 2H), 7.43 – 7.39 (m, 7H), 7.33 (m, 4H), 7.26 – 7.21 (m, 2H), 5.61 (s, 1H). ^{13}C NMR (150 MHz, $CDCl_3$) 160.9, 144.0, 136.4, 130.9, 128.7, 128.6, 128.57, 127.8, 127.1, 78.0; HRMS (ESI): Exact mass calcd for $C_{20}H_{18}N$ [M+H$^+$], 272.1439. Found 272.1454. This data is consistent with the data previously reported for compound **7o**.[9]

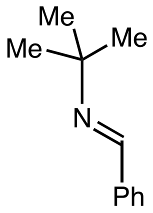

(*E*)-*N*-*tert*-Butyl-1-phenylmethanimine (7p): The reaction was carried out according to the general procedure (**B**) using 2-methylpropan-2-amine (105 µL, 1.0 mmol). A comparison of the ^{1}H NMR integrals for the imine proton of **7p** and the benzylic protons of benzyl ether indicated that **7p** was obtained in 54% yield in trial #1, and 58% yield in trial #2, for an average yield of 56%. Following purification, product **7p** was isolated as a white semi-solid. IR (film) 3060, 2966, 1641 cm^{-1}. ^{1}H NMR (600 MHz, $CDCl_3$) 8.28 (s, 1H), 7.80 – 7.71 (m, 2H), 7.48 – 7.35 (m, 3H), 1.31 (s, 9H). ^{13}C NMR (150 MHz, $CDCl_3$) 155.3, 137.3, 130.3, 128.6, 128.0, 57.4, 29.9; HRMS (ESI): Exact mass calcd for $C_{11}H_{16}N$ [M+H$^+$], 162.1283. Found 162.1285. This data is consistent with the data previously reported for compound **7p**.[10]

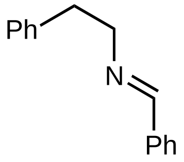

(*E*)-*N*-Phenethyl-1-phenylmethanimine (7q): The reaction was carried out according to the general procedure (**B**) using 2-phenylethan-1-amine (126 µL, 1.0 mmol). A comparison of the ^{1}H NMR integrals for the imine proton of **7q** and the benzylic protons of benzyl ether indicated that **7q** was obtained in 16% yield in trial #1, and 17% yield in trial #2, for an average yield of 17%. Following purification, product **7q** was isolated as a white solid, m.p. 33 °C. IR (film) 3024, 2920, 1645 cm^{-1}. ^{1}H NMR (600 MHz, $CDCl_3$) 8.18 (s, 1H), 7.73 – 7.67 (m, 2H), 7.45 – 7.38 (m, 3H), 7.31 – 7.27 (m, 2H), 7.26 – 7.23 (m, 2H), 7.22 – 7.18 (m, 1H), 3.87 (td, *J* = 7.5, 1.3 Hz, 2H), 3.02 (t, *J* = 7.5 Hz, 2H). ^{13}C NMR (150 MHz, $CDCl_3$) 161.6, 140.1, 136.3, 130.7, 129.2, 128.7, 128.5, 128.2, 126.2, 63.3, 37.7; HRMS (ESI): Exact mass calcd for $C_{15}H_{16}N$ [M+H$^+$], 210.1283. Found 210.1243.

---

[8] Srimani, D.; Sarkar, A.; *Tetrahedron Lett.* **2008,** *49,* 6304-6307.

[9] Krueger, C. A.; Kuntz, K. W.; Dzierba, C. D.; Wirschun, W. G.; Gleason, J. D.; Snapper, M. L.; Hoveyda, A. H.; *J. Am. Chem. Soc.* **1999,** *121,* 4284-4285.

[10] Guzen, K. P.; Guarezemini, A. S.; Orfao, A. T. G.; Cella, R.; Pereira, C. M. P.; Stefani, H. A.; *Tetraghedron Lett.* **2007,** *48,* 1845-1848.

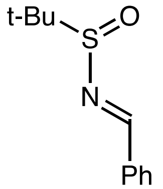

(*E*)-*N*-Benzylidene-2-methylpropane-2-sulfinamide (7t): The reaction was carried out according to the general procedure (**B**) using 2-methylpropane-2-sulfinamide (121.2 mg, 1.0 mmol). A comparison of the ^{1}H NMR integrals for the imine proton of **7t** and the benzylic protons of benzyl ether indicated that **7t** was obtained in 21% yield in trial #1, and 24% yield in trial #2, for an average yield of 22%. Following purification, product **7t** was isolated as a pale-yellow liquid. IR (film) 3060, 2958, 1606, 1085cm^{-1}. ^{1}H NMR (600 MHz, $CDCl_3$) 8.59 (s, 1H), 7.89 – 7.78 (m, 2H), 7.52 – 7.50 (m, 1H), 7.49 – 7.45 (m, 2H), 1.27 (s, 9H).^{13}C NMR (150 MHz, $CDCl_3$) 162.9, 134.2, 132.5, 129.5, 129.1, 57.9, 22.7; HRMS (ESI): Exact mass calcd for $C_{11}H_{16}NOS$ [M+H$^+$], 210.0953. Found 210.0956. This data is consistent with the data previously reported for compound **7t**.[11]

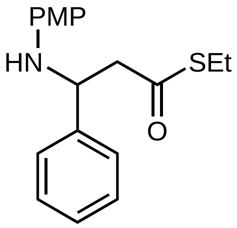

***S*-Ethyl 3-((4-methoxyphenyl)amino)-3-phenylpropanethioate (11)**: In a manner closely related to an analogous procedure[12], to a solution of 2,6-di-*tert*-butyl-1,4-benzoquinone **2c** (0.1 mmol, 22.0 mg, 0.2 equiv) and p-anisidine (**6a**, 1.0 mmol, 123 mg, 2.0 equiv) in ethanol (1.6 mL) was added 2-phenylglycinol **1a** (68.6 mg, 0.5 mmol). The vial was purged with O_2 and then heated to 70 °C with vigorous stirring for 24 h. The ethanol was removed by rotary evaporation and the oxidative deformylation product was further pumped for 0.5h with a high-vacuum pump. The remaining mixture was dissolved in dry THF (1.6 mL, 0.312 M) and the solution was cooled to 0 °C. To this solution was added tetrafluoroboric acid solution (48 wt. % in H_2O, 90.0 μL, 0.75 mmol) and silyl ketene (thio)acetal[13] (1.5 mmol). The resulting solution was allowed to stir for 2h under argon at 0 °C. Sodium phosphate buffer (PH = 7, 2 mL) was added followed by saturated aq $NaHCO_3$ (0.5 mL). The mixture was transferred to a separatory funnel by aid of Et_2O, shaken vigorously and the organic phase was collected. The aqueous phase was further extracted with Et_2O (3 x 5.0 mL) and the combined organic extracts were dried over Na_2SO_4, filtered and concentrated under reduced pressure. Flash chromatography on triethylamine treated silica gel (10% ethyl acetate in hexanes) provided the product **11** (93.3 mg, 60%) as a yellow solid, m.p. 76 °C. IR (film) 3396, 3030, 2929, 1676 cm^{-1}. ^{1}H NMR (500 MHz, $CDCl_3$) 7.38 – 7.35 (m, 2H), 7.34 – 7.30 (m, 2H), 7.26 – 7.22 (m, Ar H, obscured by $CDCl_3$), 6.71 – 6.65 (m, 2H), 6.53 – 6.48 (m, 2H), 4.76 (t, J = 6.7 Hz, 1H), 4.27 (s, 1H), 3.69 (s, 3H), 2.97 (d, J = 6.7 Hz, 2H), 2.86 (qd, J = 7.4, 0.8 Hz, 2H), 1.21 (t, J = 7.5 Hz, 3H). ^{13}C NMR (126 MHz, $CDCl_3$) 197.6, 152.4, 142.4, 141.1, 128.9, 127.6, 126.4, 115.2, 114.8, 56.7, 55.8, 51.8, 23.8, 14.7; HRMS (ESI): Exact mass calcd for $C_{18}H_{21}NO_2S$ [M+H$^+$], 316.1371. Found 316.1383. This data is consistent with the data previously reported for compound **11**.[13]

---

[11] Vyas, D. J.; Frohlich, R.; Oestreich, M.; *Org. Lett.* **2011,** *13,* 2094-2097.

[12] Haugeberg, B. H.; Phan, J. H.; Liu, X.; O'Connor, T. J.; Clift, M. D.; *Chem. Commun.* **2017,** *53,* 3062-3065.

[13] Prepared according to: Chua, S. S.; Alni, A.; Chan, L. T. J.; Yamane, M.; Loh T. P. *Tetrahedron*, **2011**, *67*, 5079.

Part 4: ^{1}H and ^{13}C NMR spectra:

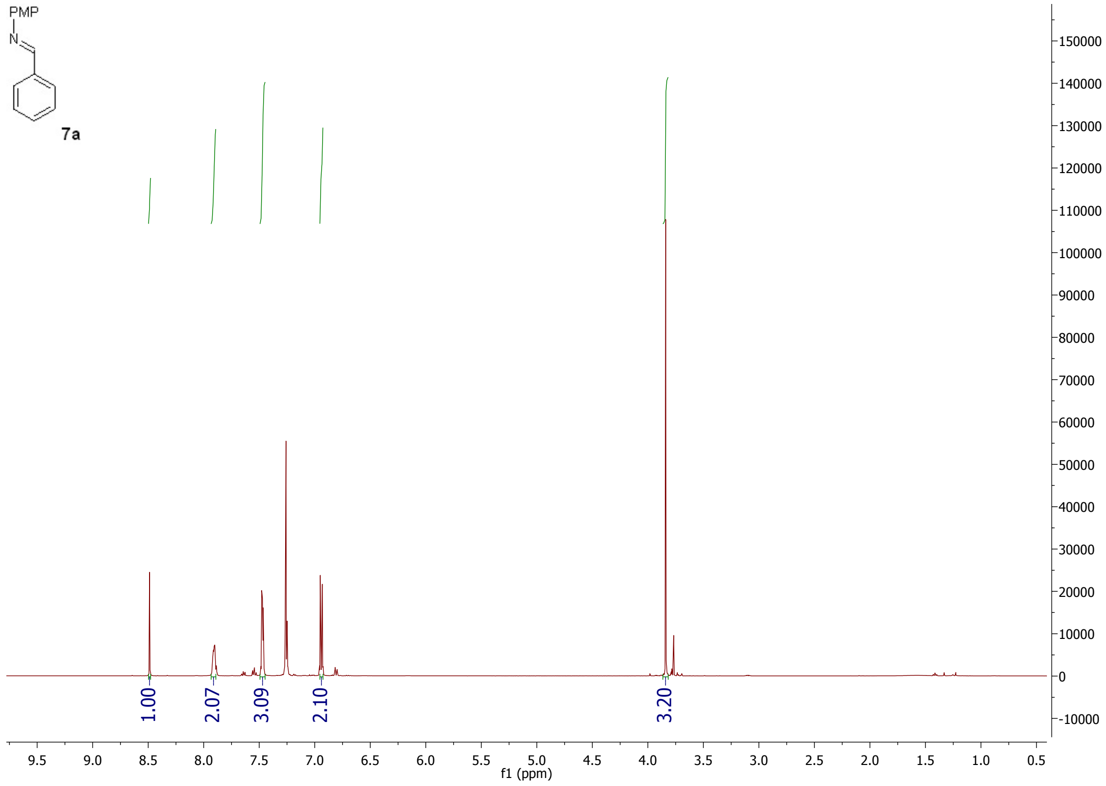

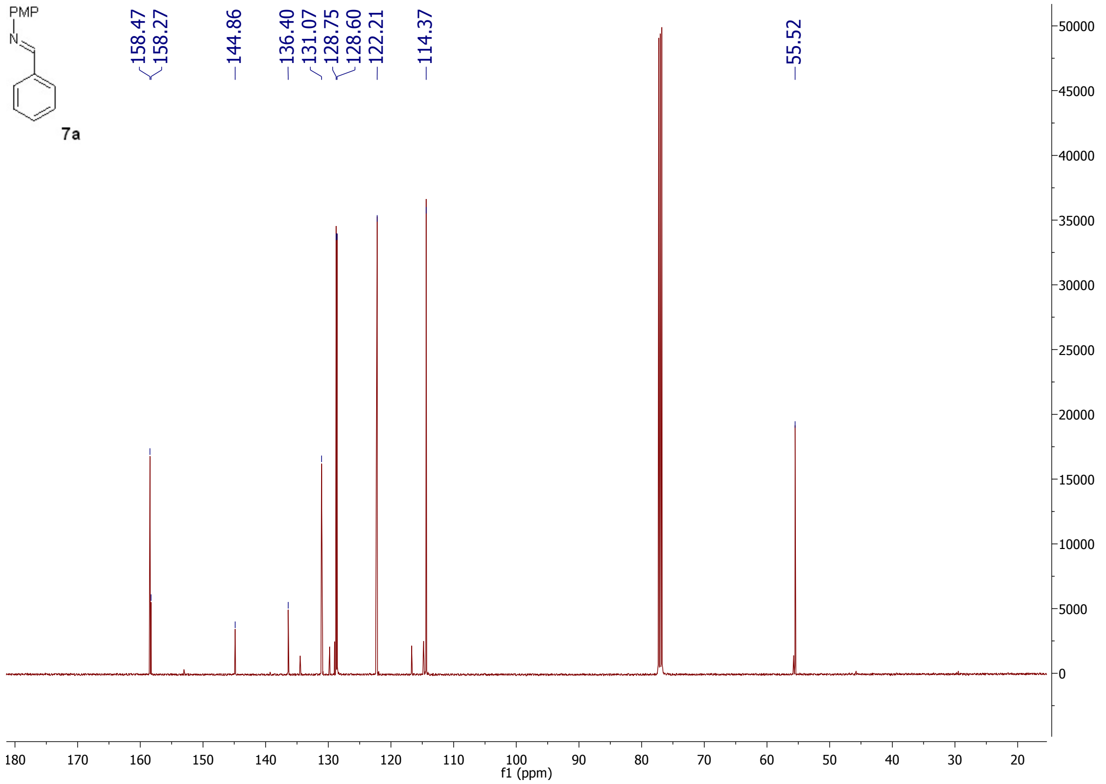

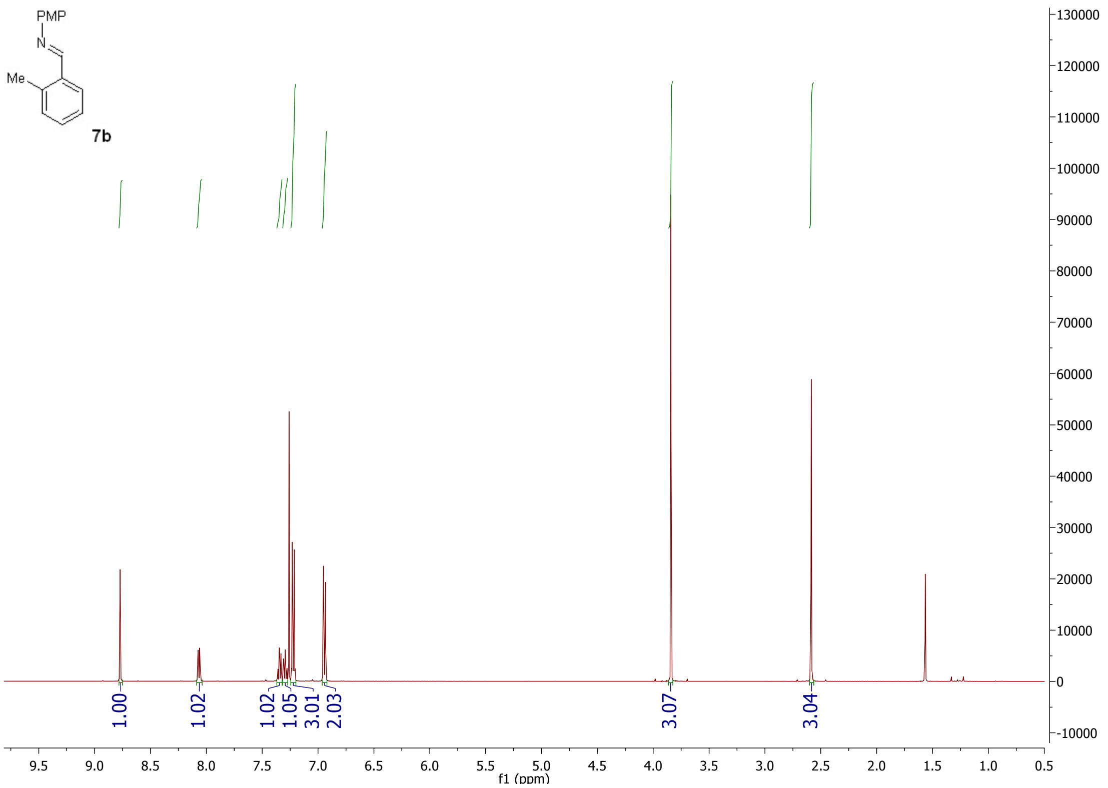
PMP
N
Me
7b
1.00
1.02
1.02
1.05
3.01
2.03
3.07
3.04
f1 (ppm)

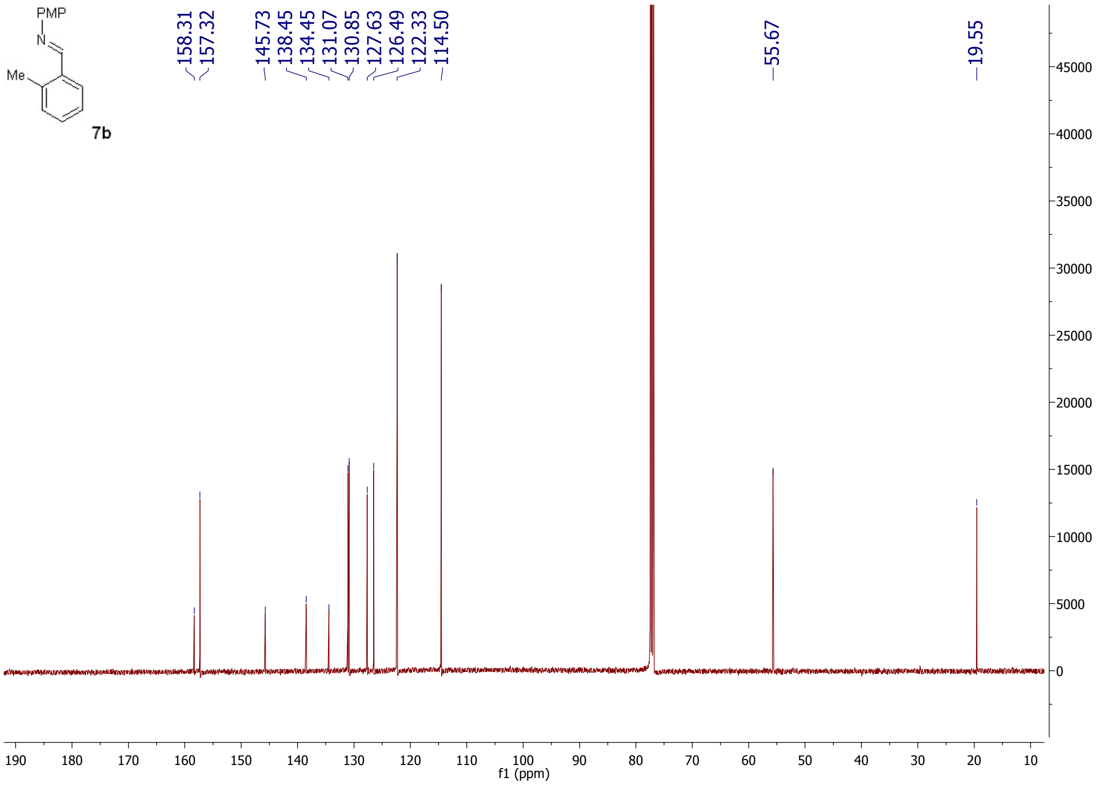
PMP
N
Me
7b
158.31
157.32
145.73
138.45
134.45
131.07
130.85
127.63
126.49
122.33
114.50
55.67
19.55
f1 (ppm)

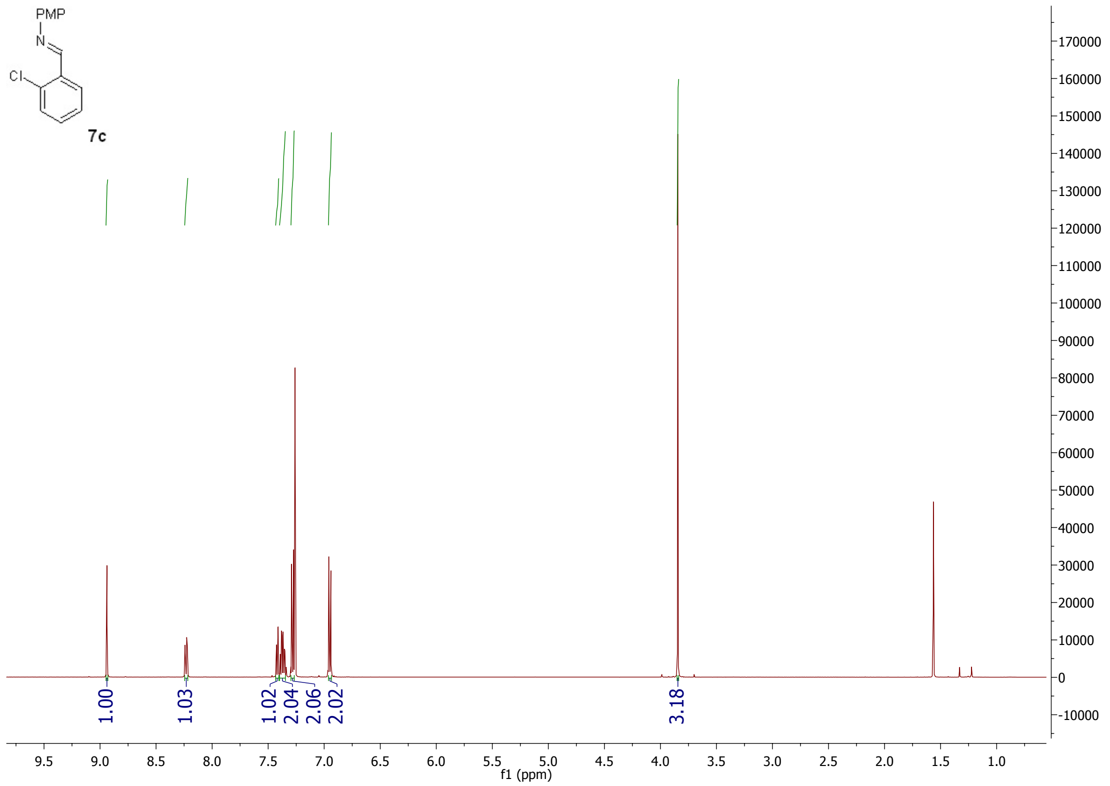
PMP
N
Cl
7c
1.00
1.03
1.02
2.04
2.06
2.02
3.18
f1 (ppm)

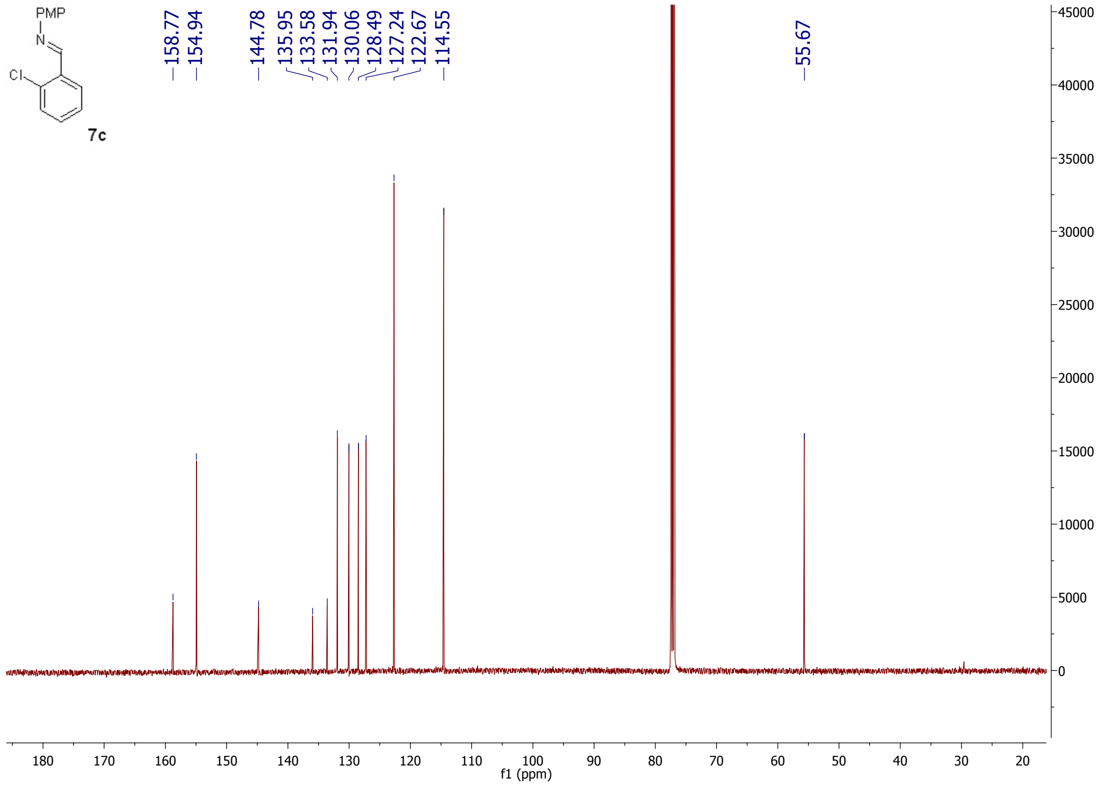
PMP
N
Cl
7c
158.77
154.94
144.78
135.95
133.58
131.94
130.06
128.49
127.24
122.67
114.55
55.67
f1 (ppm)

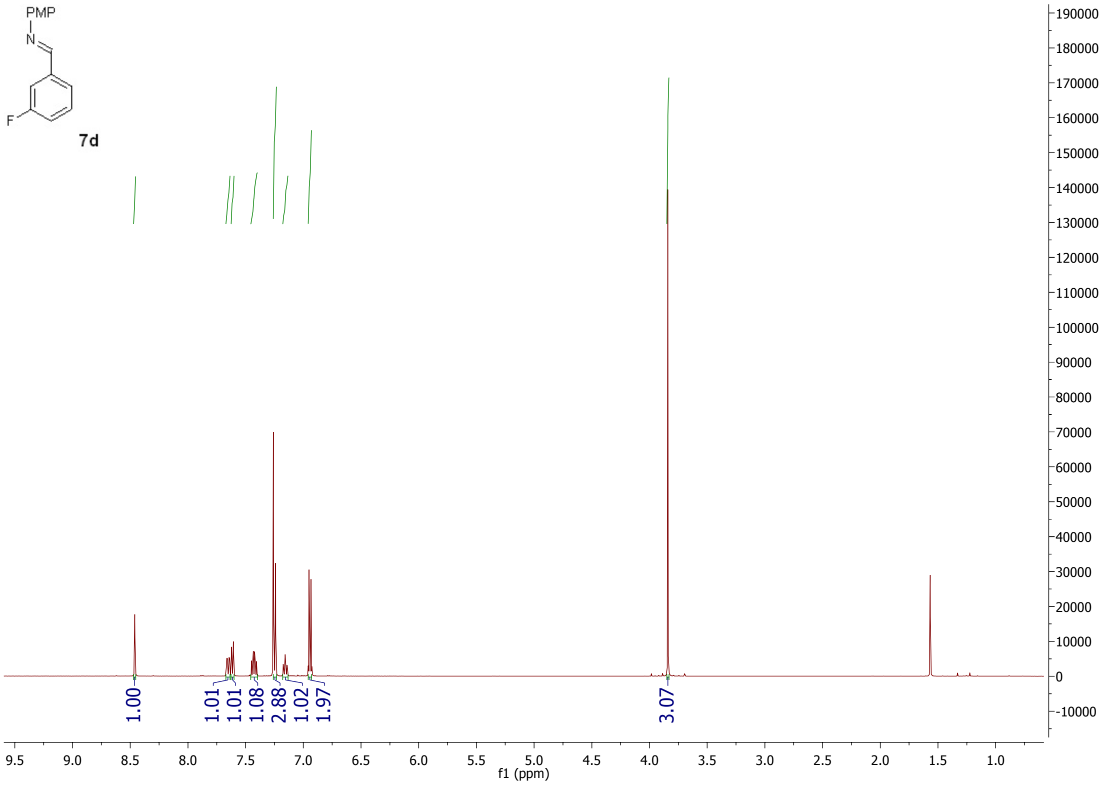
PMP
N
F
7d
1.00
1.01
1.01
1.08
2.88
1.02
1.97
3.07
f1 (ppm)

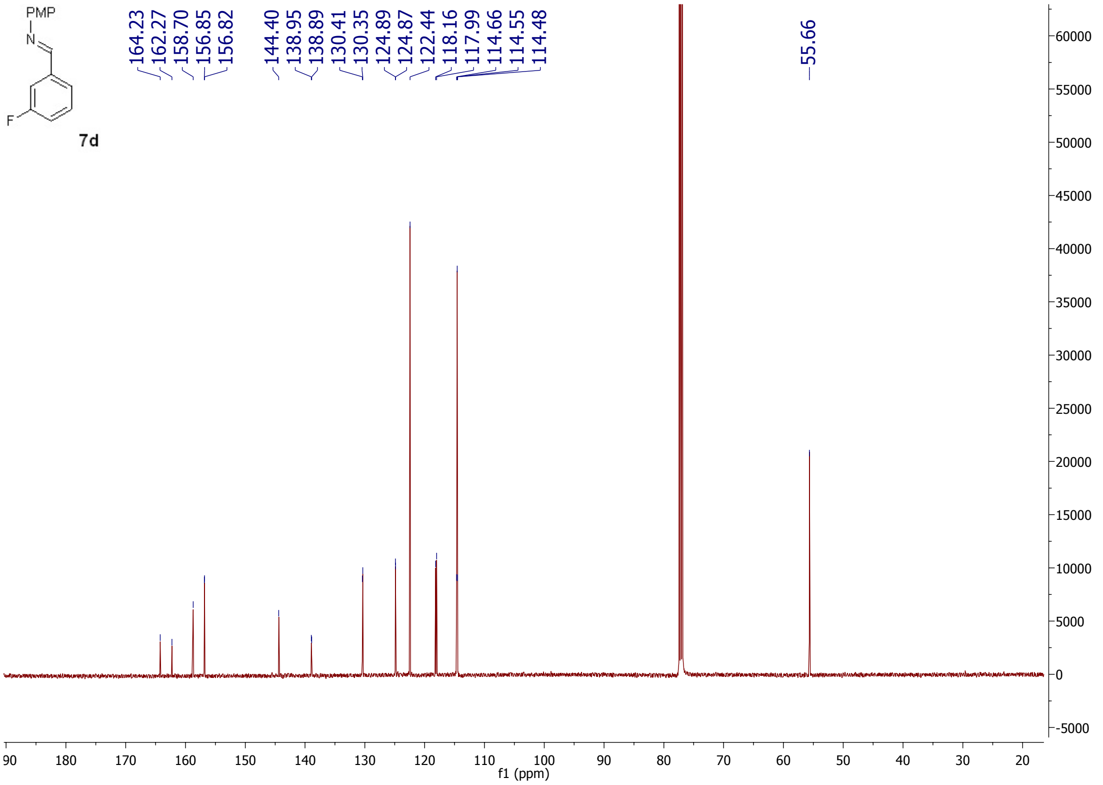
PMP
N
F
7d
164.23
162.27
158.70
156.85
156.82
144.40
138.95
138.89
130.41
130.35
124.89
124.87
122.44
118.16
117.99
114.66
114.55
114.48
55.66
f1 (ppm)

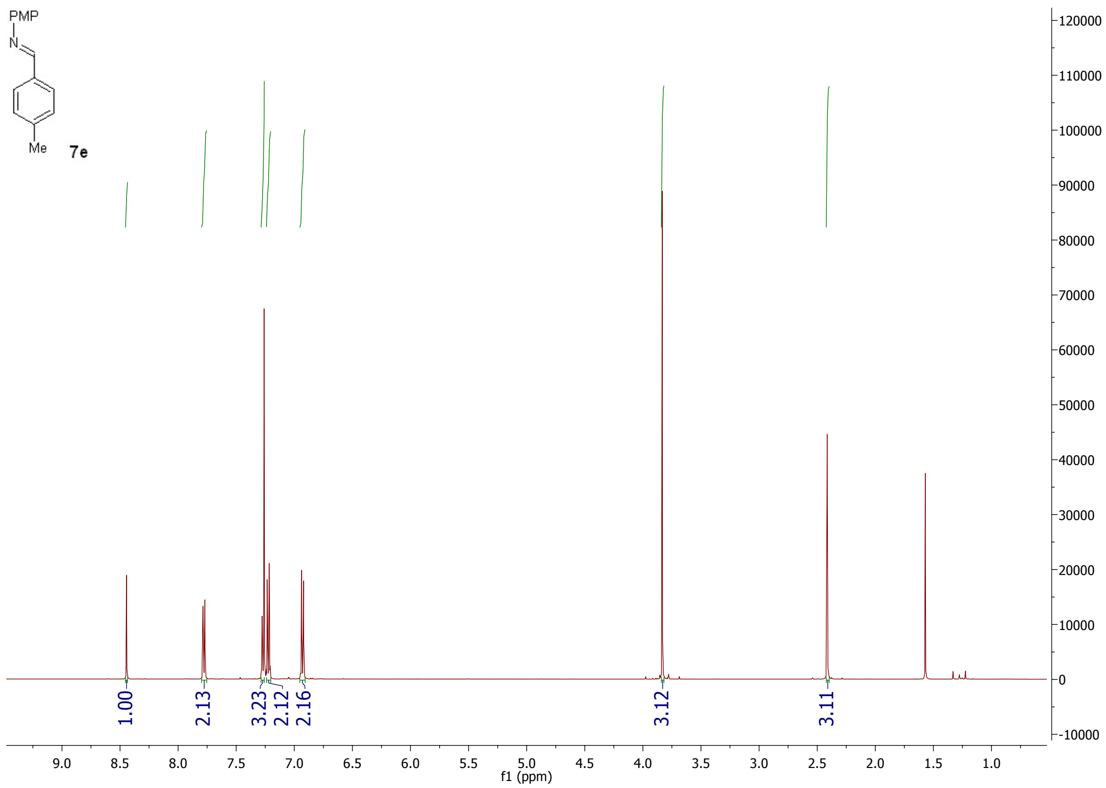
PMP
N
Me
7e
1.00
2.13
3.23
2.12
2.16
3.12
3.11
f1 (ppm)

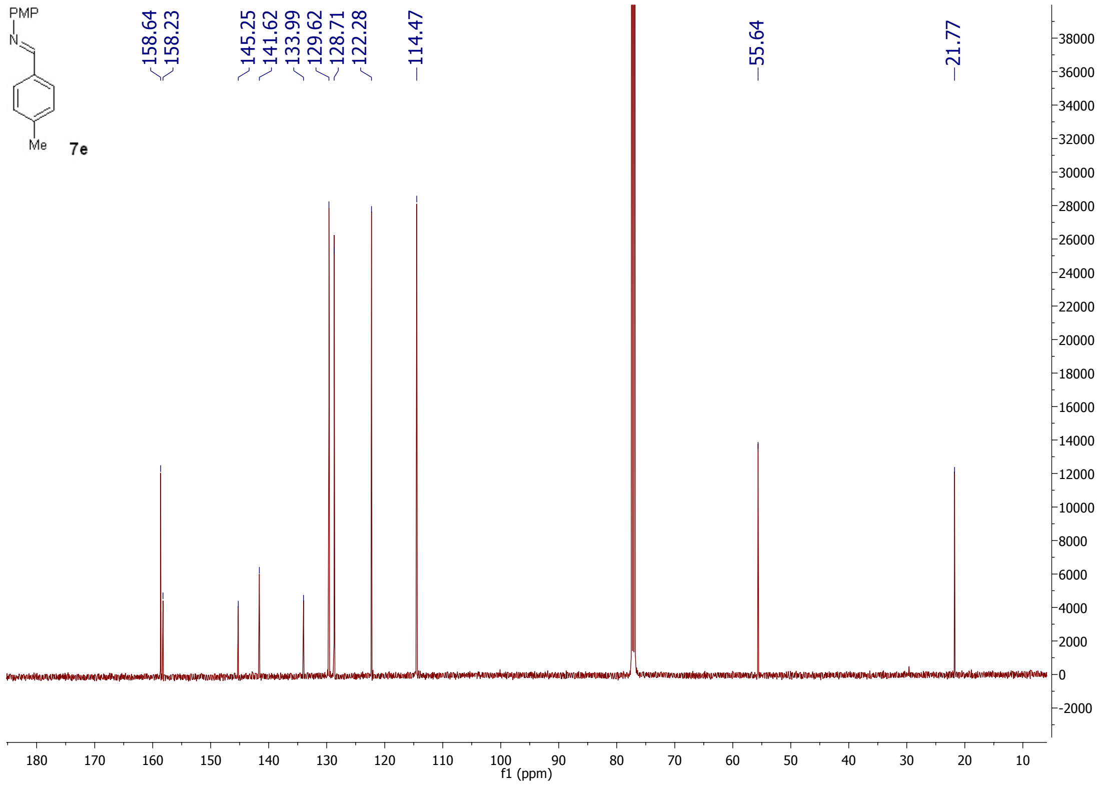
PMP
N
Me
7e
158.64
158.23
145.25
141.62
133.99
129.62
128.71
122.28
114.47
55.64
21.77
f1 (ppm)

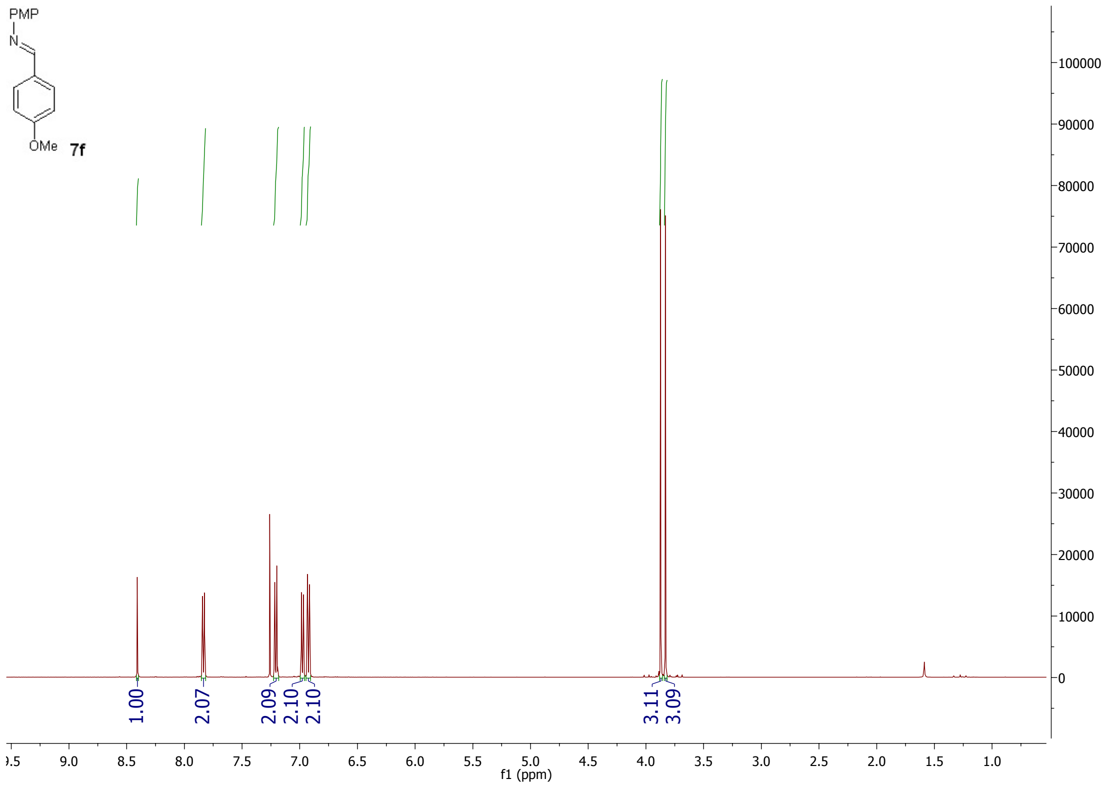
PMP
N
OMe
7f
1.00
2.07
2.09
2.10
2.10
3.11
3.09
f1 (ppm)

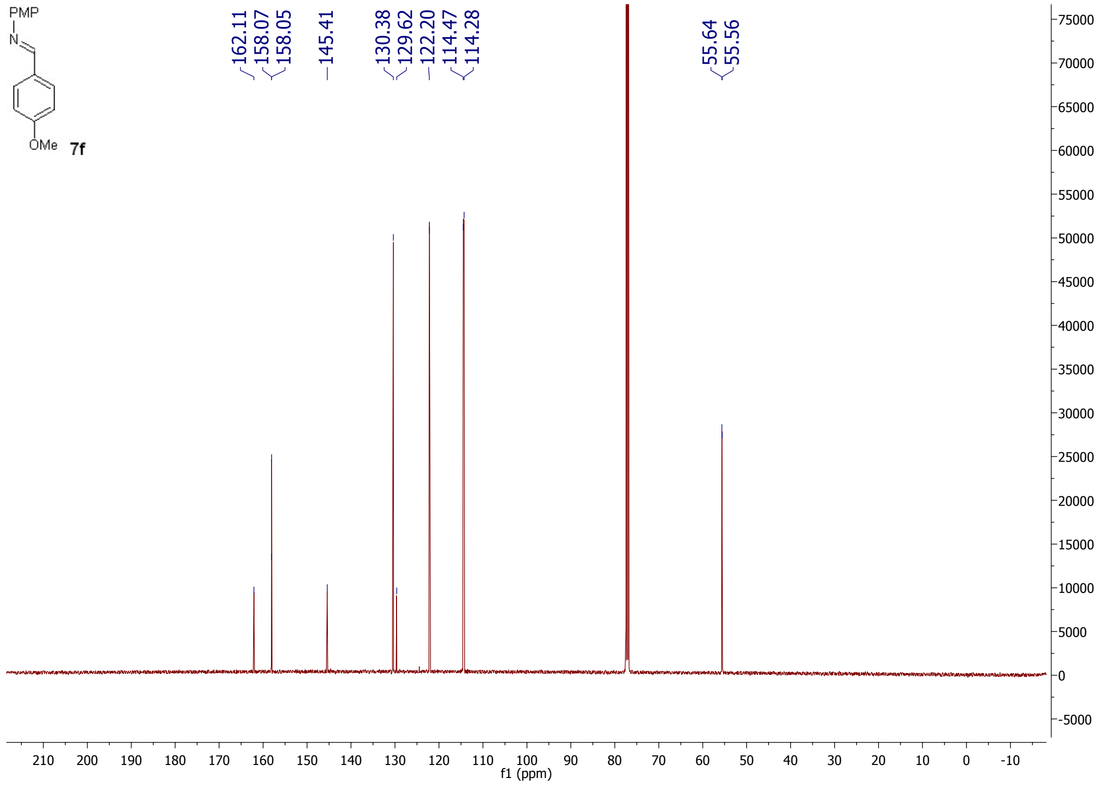
PMP
N
OMe
7f
162.11
158.07
158.05
145.41
130.38
129.62
122.20
114.47
114.28
55.64
55.56
f1 (ppm)

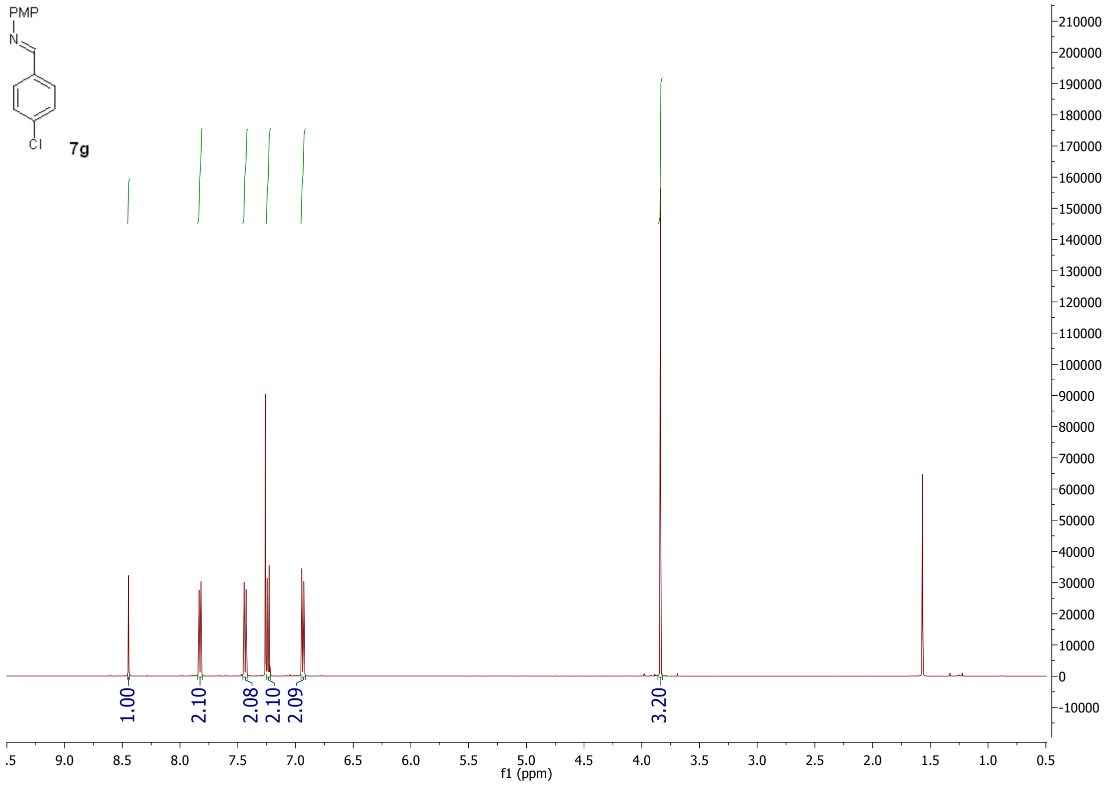
PMP
N
Cl
7g
1.00
2.10
2.08
2.10
2.09
3.20
f1 (ppm)

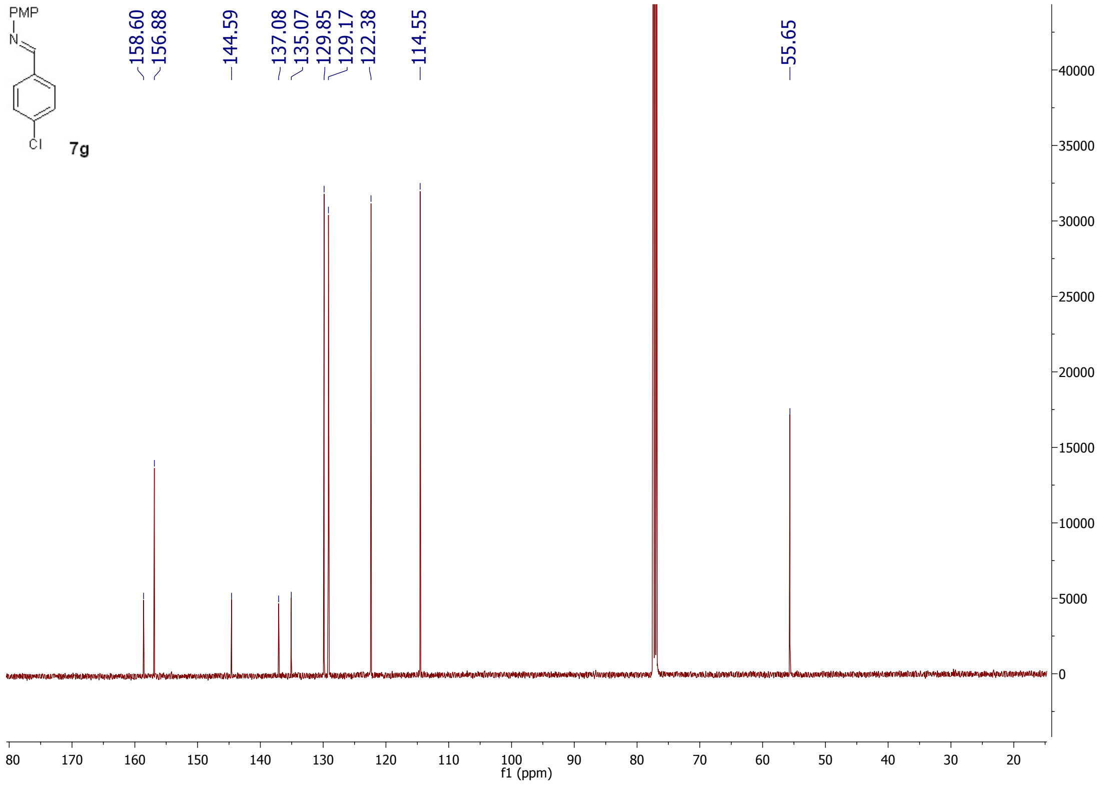
PMP
N
Cl
7g
158.60
156.88
144.59
137.08
135.07
129.85
129.17
122.38
114.55
55.65
f1 (ppm)

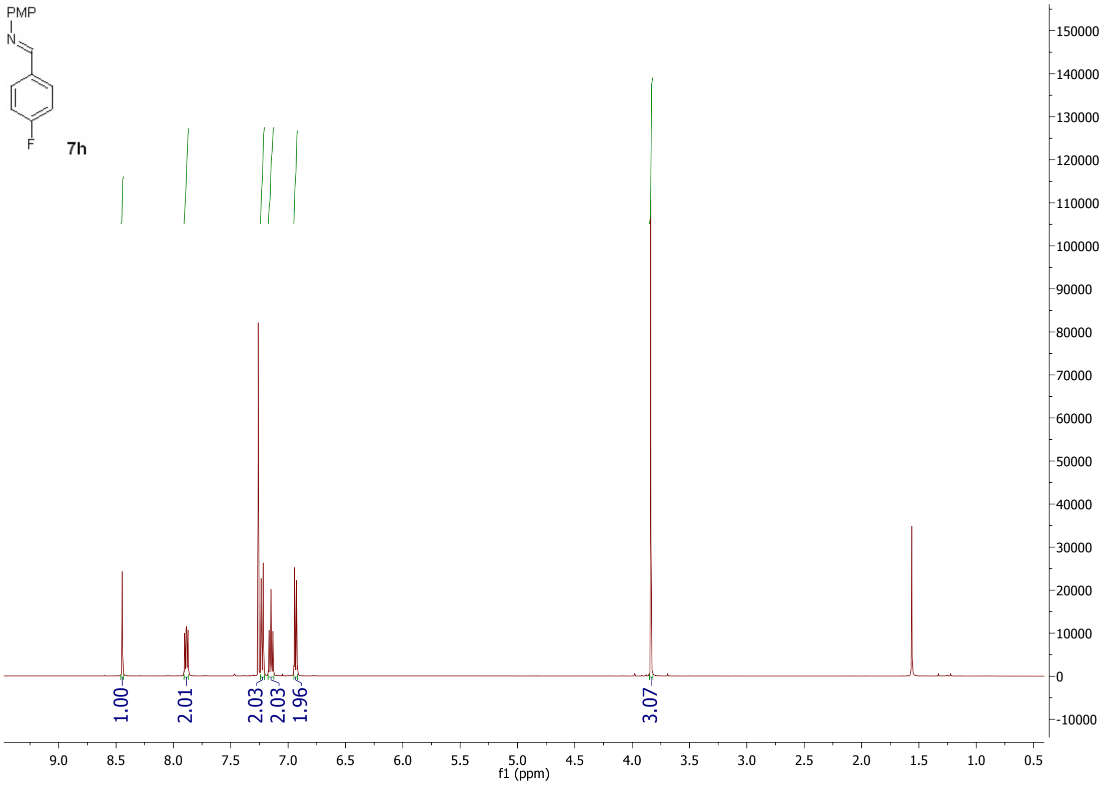
PMP
N
F
7h
1.00
2.01
2.03
2.03
1.96
3.07
f1 (ppm)

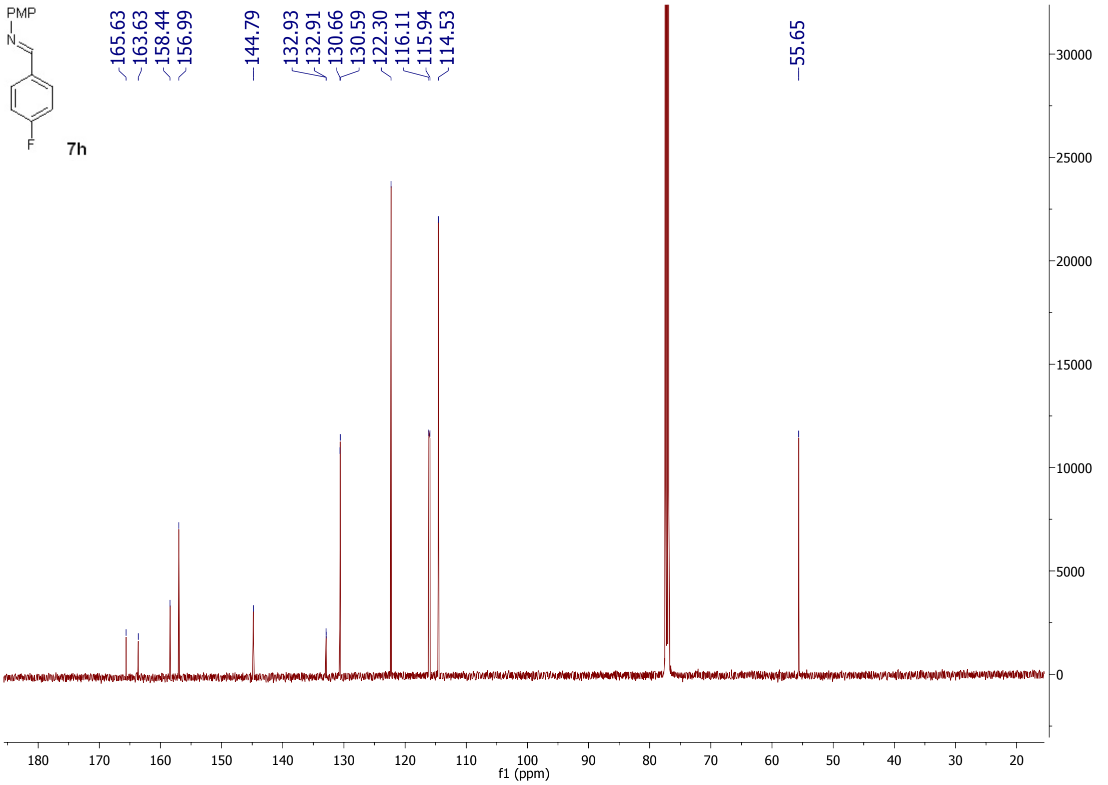
PMP
N
F
7h
165.63
163.63
158.44
156.99
144.79
132.93
132.91
130.66
130.59
122.30
116.11
115.94
114.53
55.65
f1 (ppm)

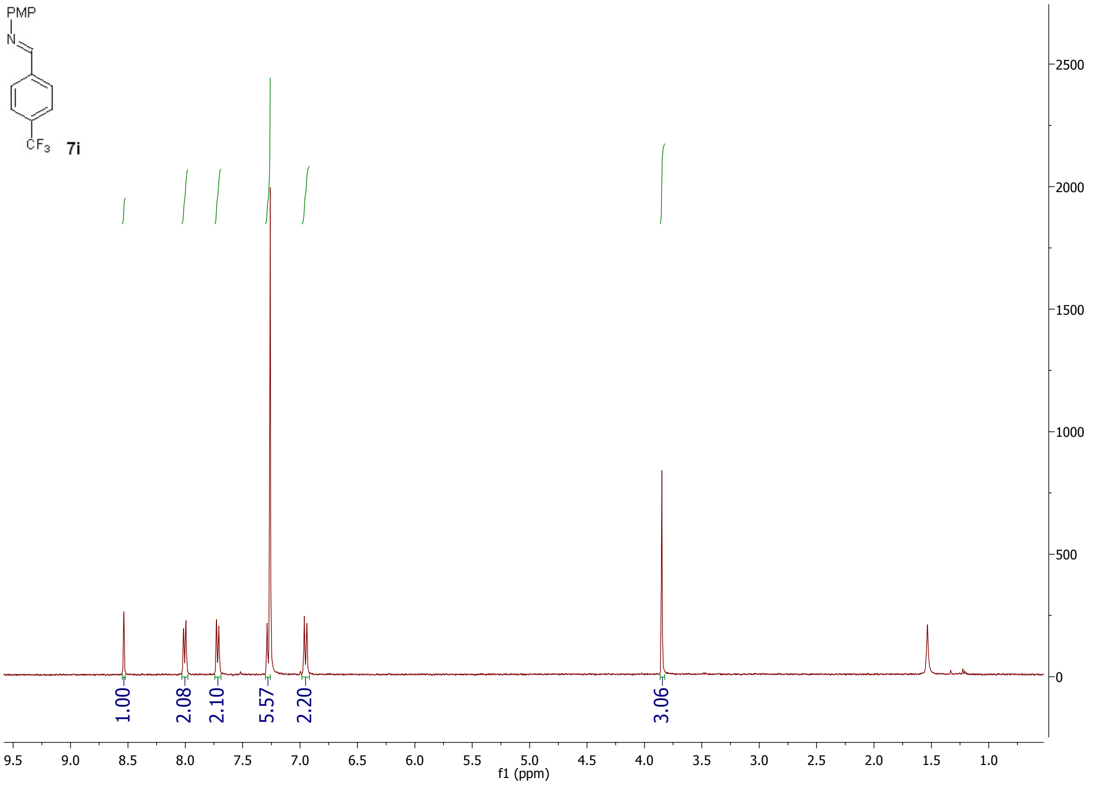
PMP
N
CF3
7i
1.00
2.08
2.10
5.57
2.20
3.06
f1 (ppm)

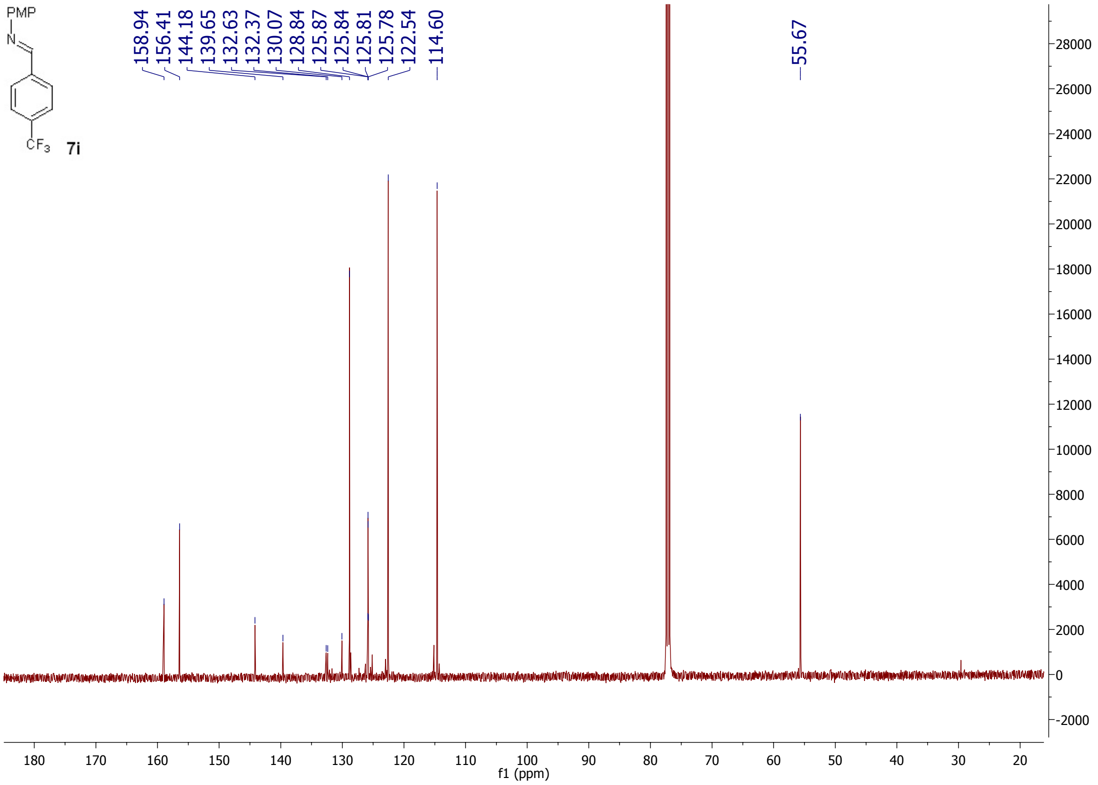
PMP
N
CF3
7i
158.94
156.41
144.18
139.65
132.63
132.37
130.07
128.84
125.87
125.84
125.81
125.78
122.54
114.60
55.67
f1 (ppm)

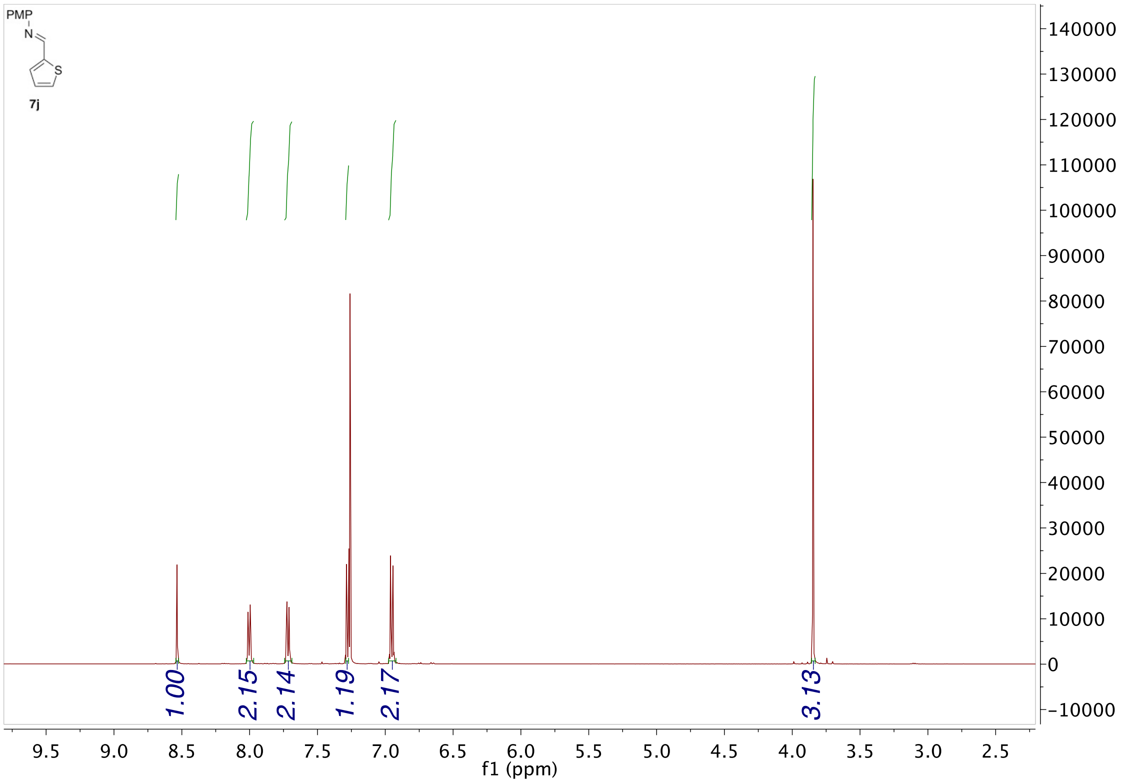
PMP
N
S
7j
1.00
2.15
2.14
1.19
2.17
3.13
f1 (ppm)

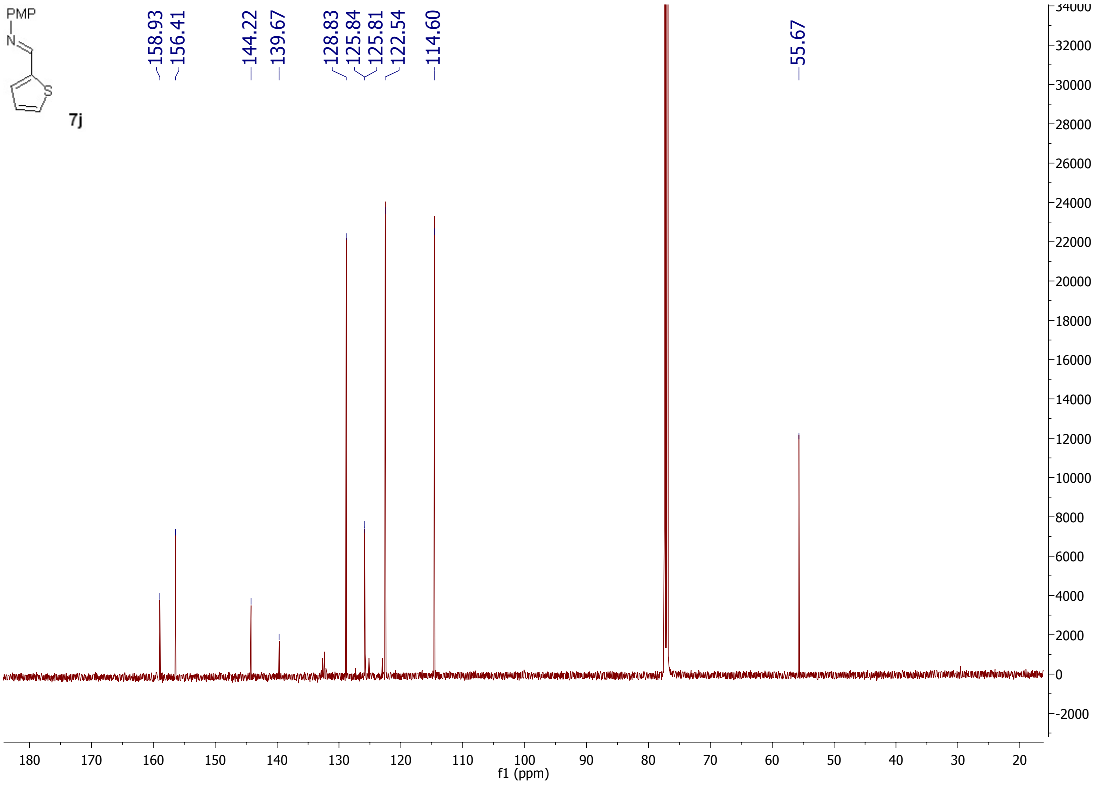
PMP
N
S
7j
158.93
156.41
144.22
139.67
128.83
125.84
125.81
122.54
114.60
55.67
f1 (ppm)

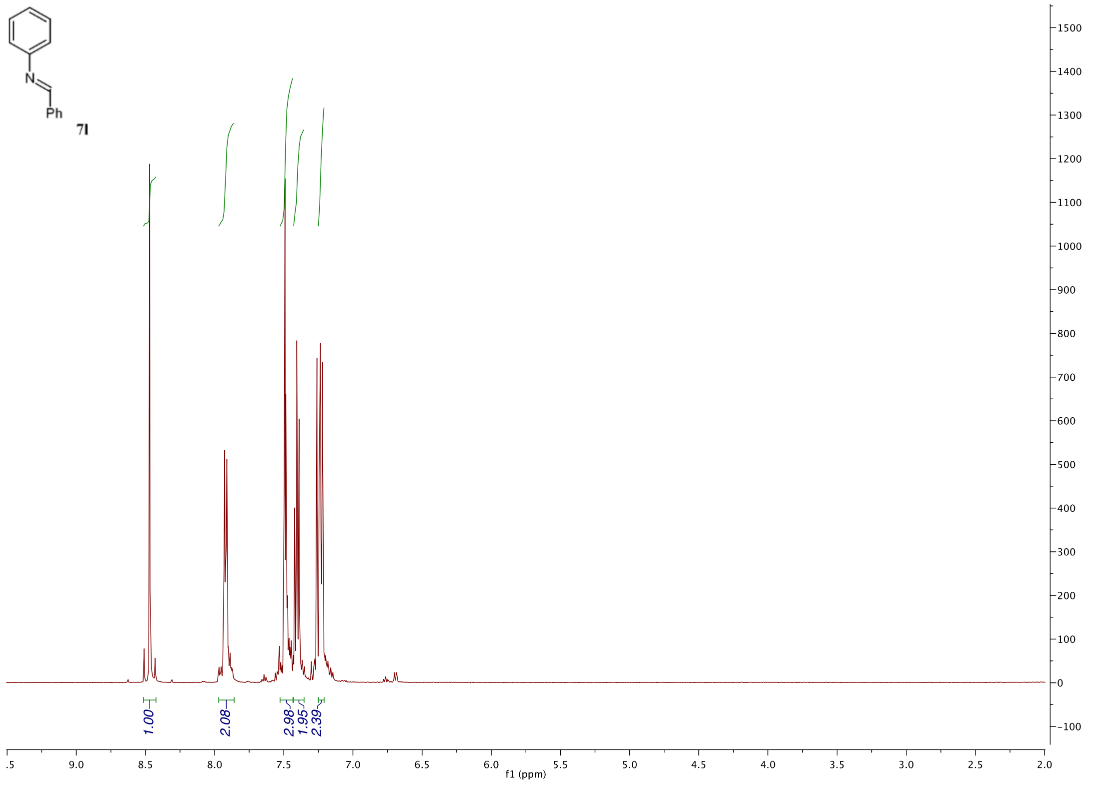
7l
1.00
2.08
2.98
1.95
2.39
f1 (ppm)

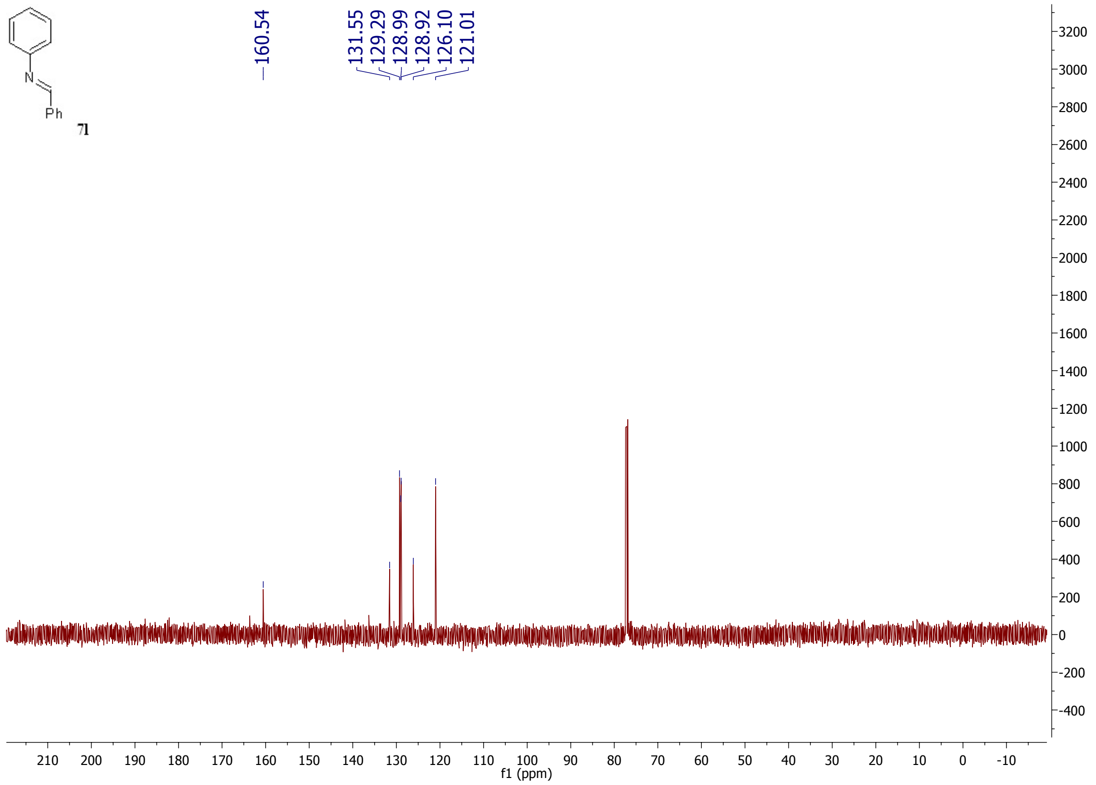
7l
160.54
131.55
129.29
128.99
128.92
126.10
121.01
f1 (ppm)

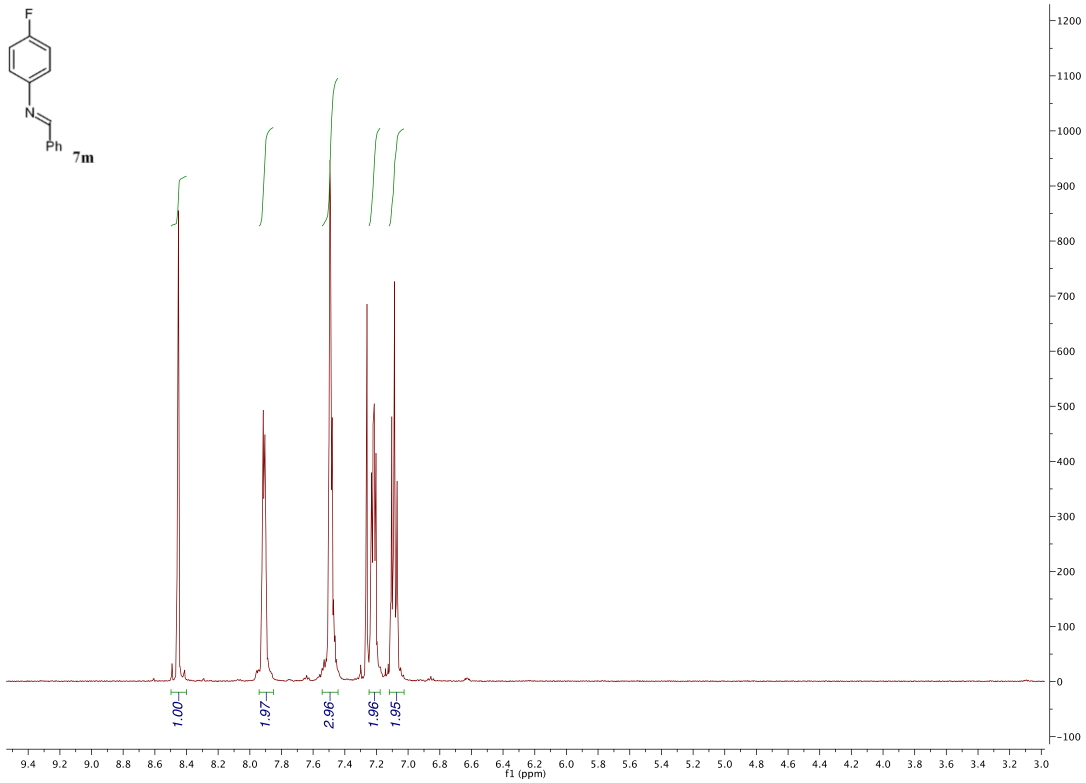
F
N
Ph
7m
1.00
1.97
2.96
1.96
1.95
f1 (ppm)

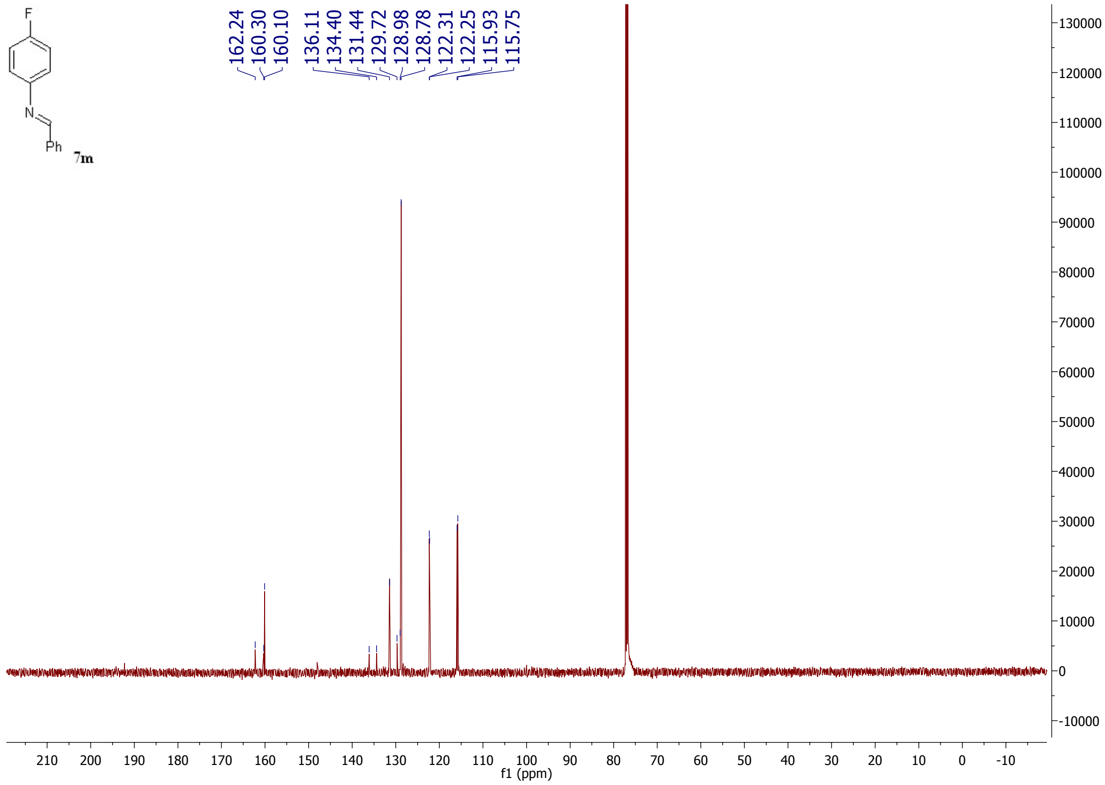
F
N
Ph
7m
162.24
160.30
160.10
136.11
134.40
131.44
129.72
128.98
128.78
122.31
122.25
115.93
115.75
f1 (ppm)

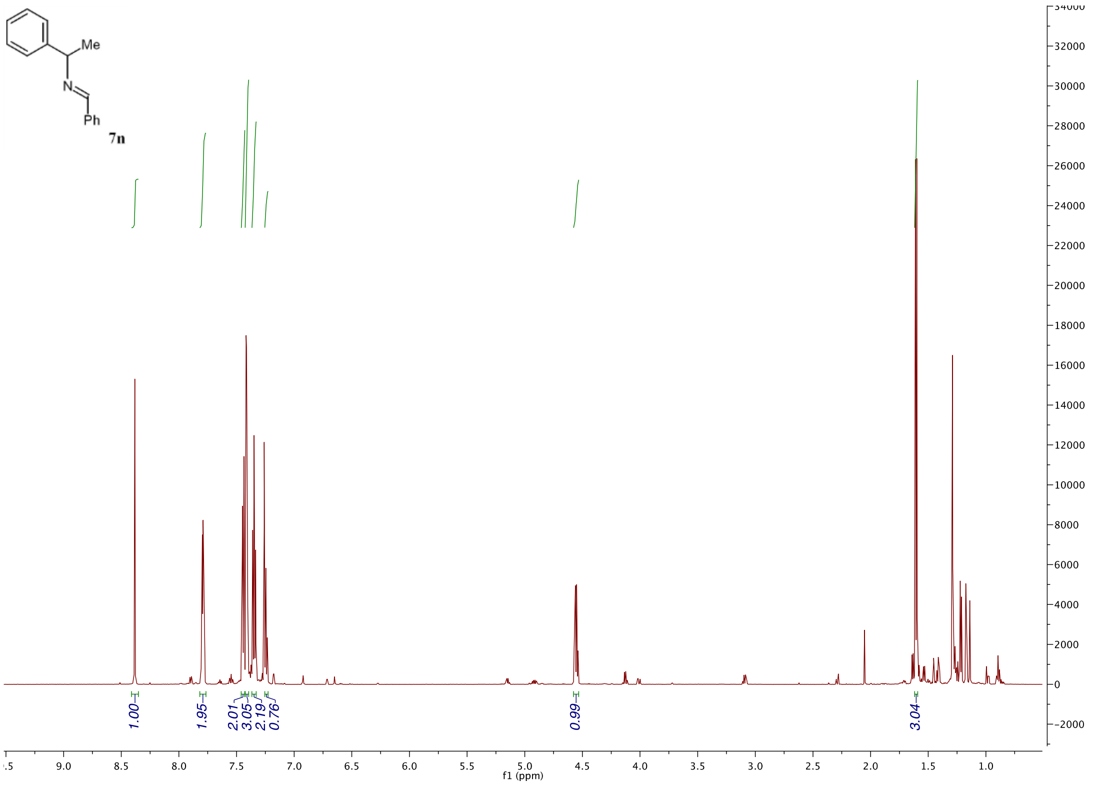
Me
N
Ph
7n
1.00
1.95
2.01
3.05
2.19
0.76
0.99
3.04
f1 (ppm)

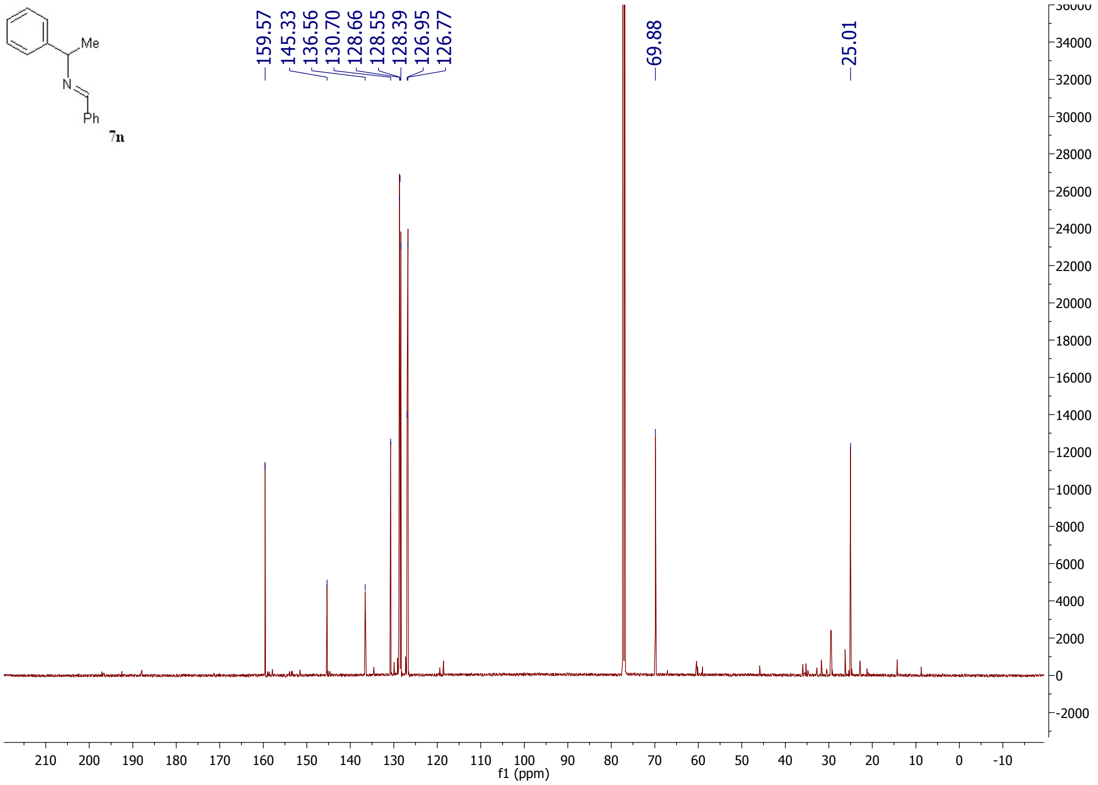
Me
N
Ph
7n
159.57
145.33
136.56
130.70
128.66
128.55
128.39
126.95
126.77
69.88
25.01
f1 (ppm)

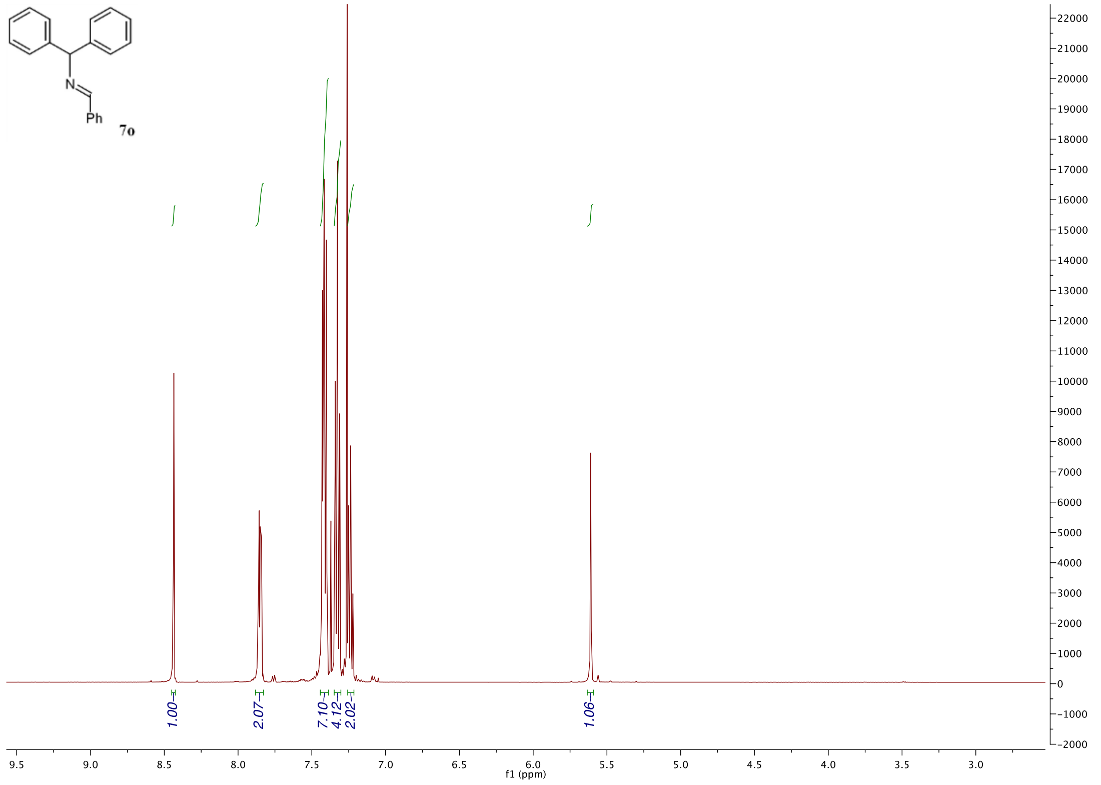
N
Ph
7o
1.00
2.07
7.10
4.12
2.02
1.06
f1 (ppm)

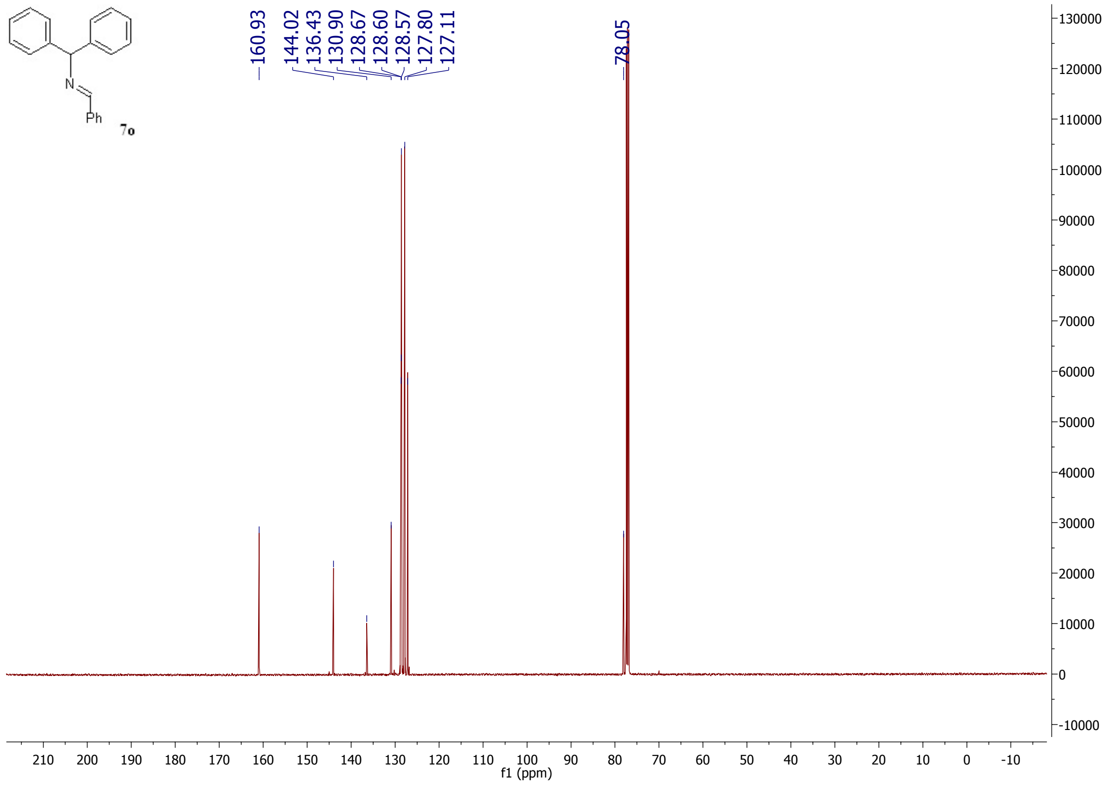
N
Ph
7o
160.93
144.02
136.43
130.90
128.67
128.60
128.57
127.80
127.11
78.05
f1 (ppm)

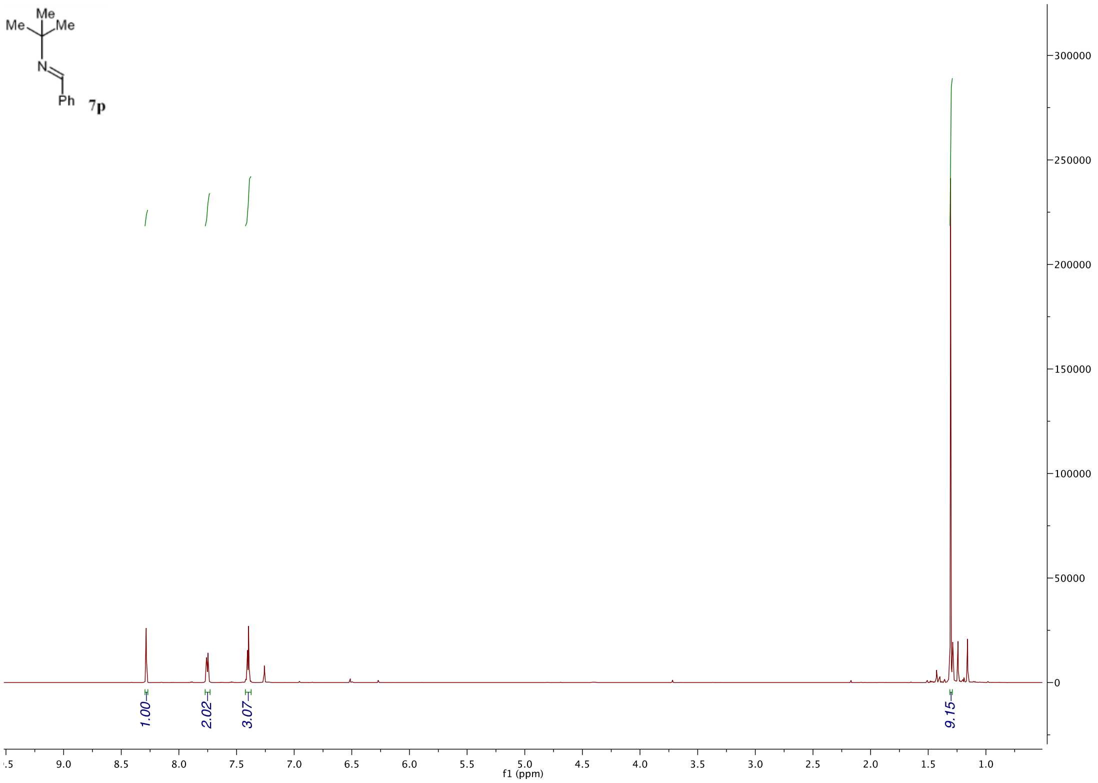
Me
Me
Me
N
Ph
7p
1.00
2.02
3.07
9.15
f1 (ppm)

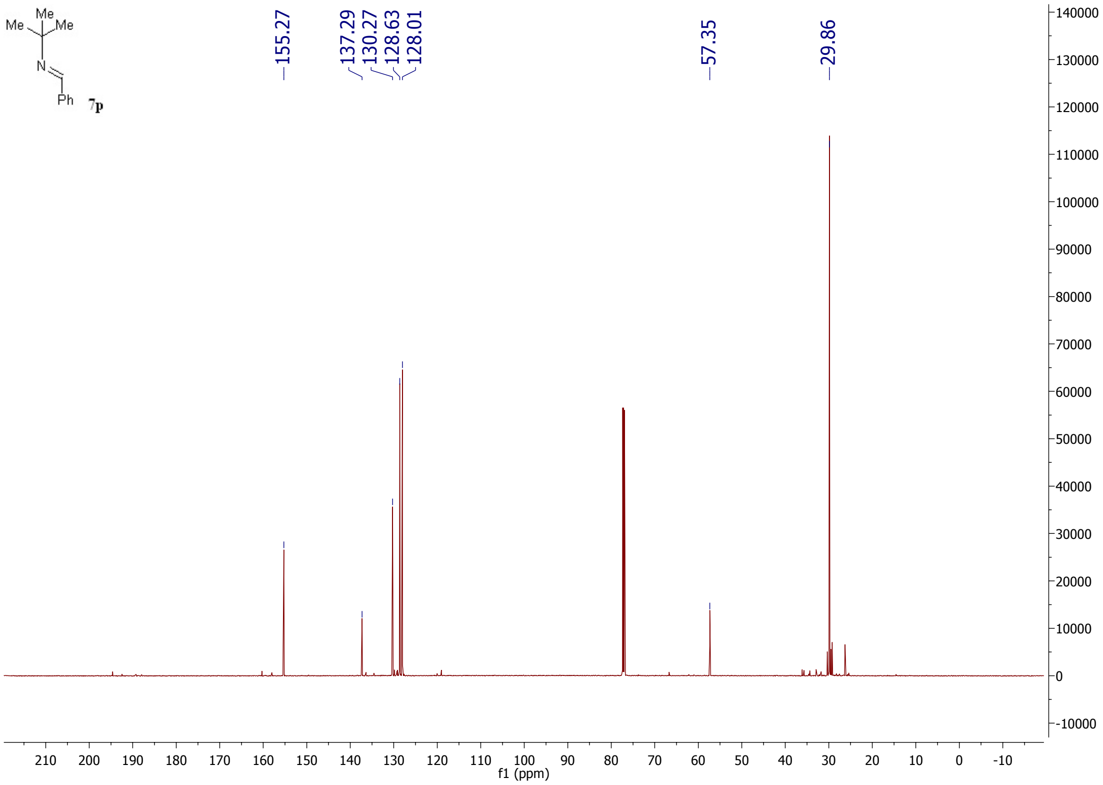
Me
Me
Me
N
Ph
7p
155.27
137.29
130.27
128.63
128.01
57.35
29.86
f1 (ppm)

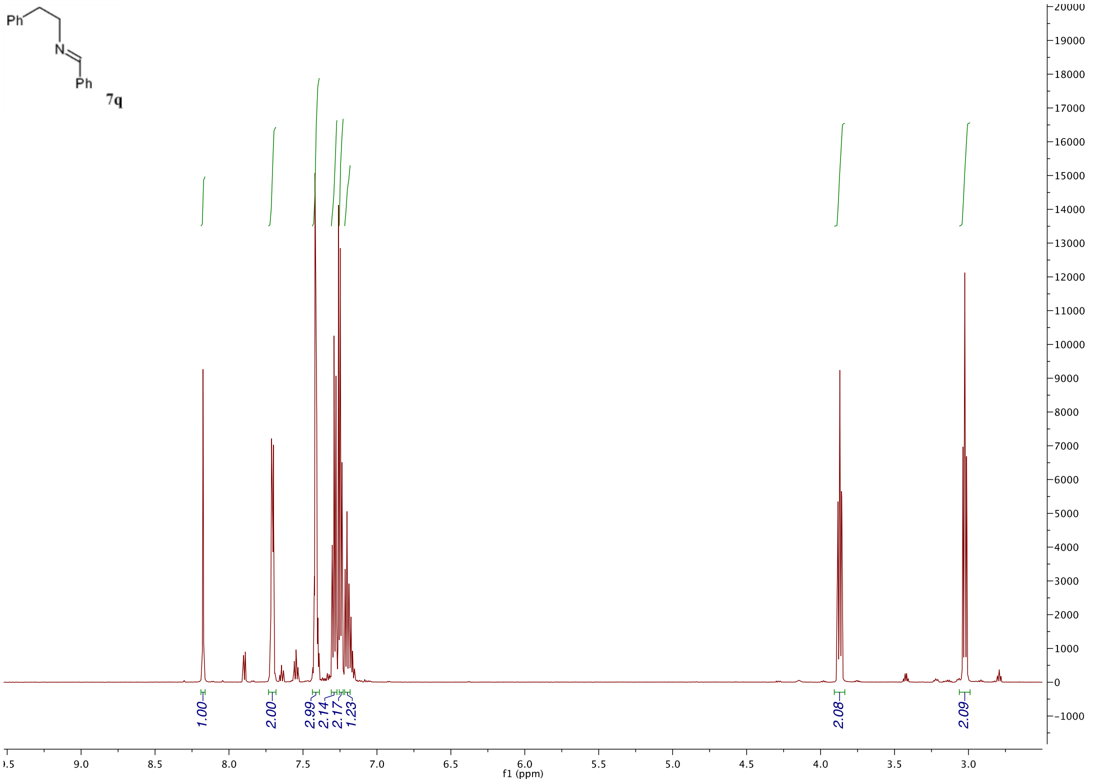
Ph
N
Ph
7q
1.00
2.00
2.99
2.14
2.17
1.23
2.08
2.09
f1 (ppm)

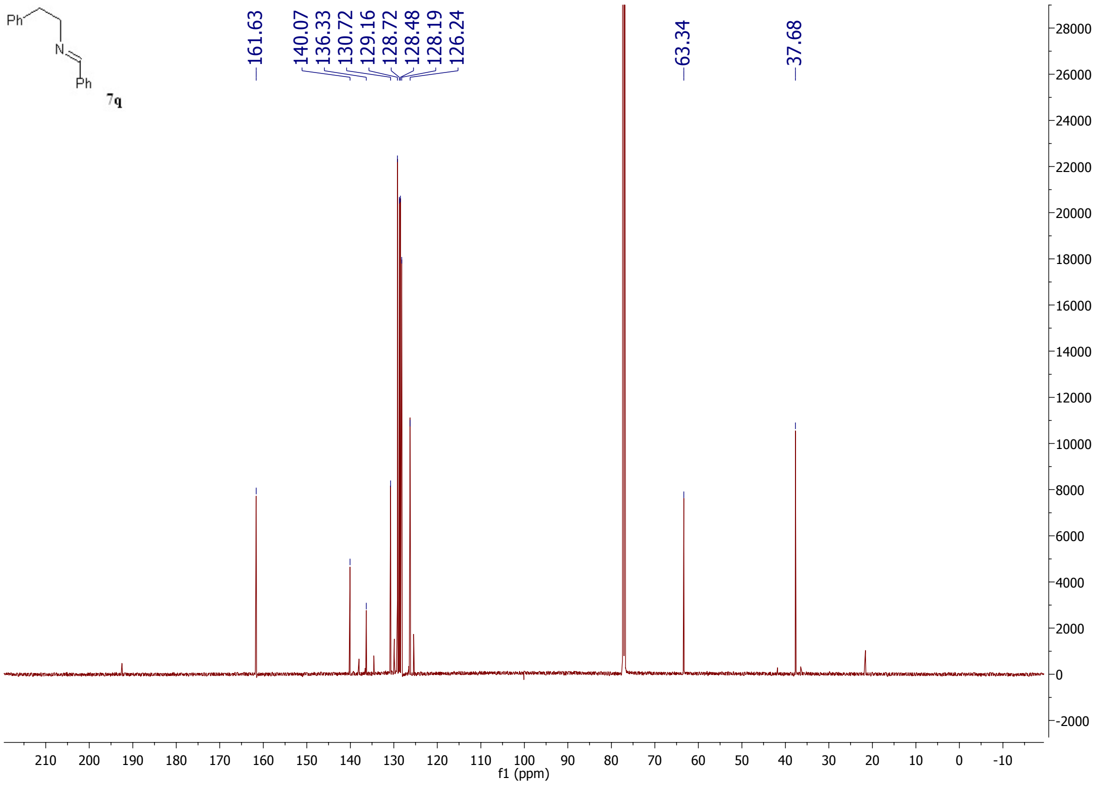
Ph
N
Ph
7q
161.63
140.07
136.33
130.72
129.16
128.72
128.48
128.19
126.24
63.34
37.68
f1 (ppm)

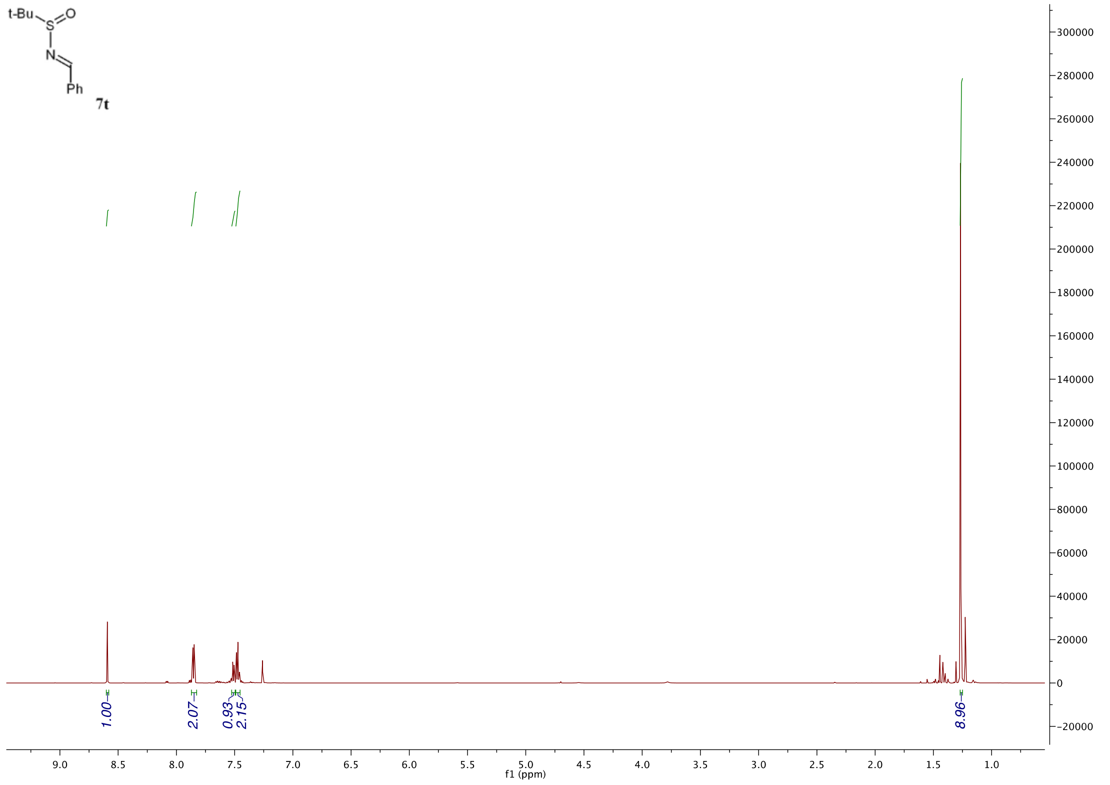
t-Bu
S
O
N
Ph
7t
1.00
2.07
0.93
2.15
8.96
f1 (ppm)

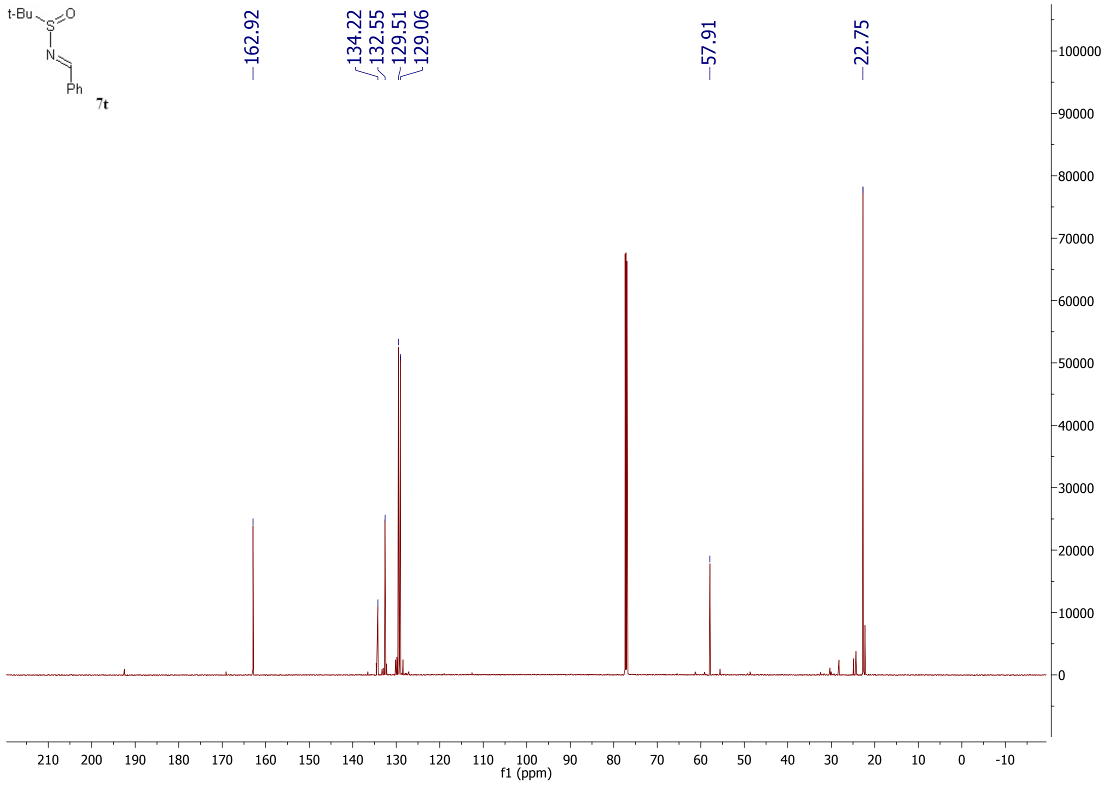
t-Bu
S
O
N
Ph
7t
162.92
134.22
132.55
129.51
129.06
57.91
22.75
f1 (ppm)

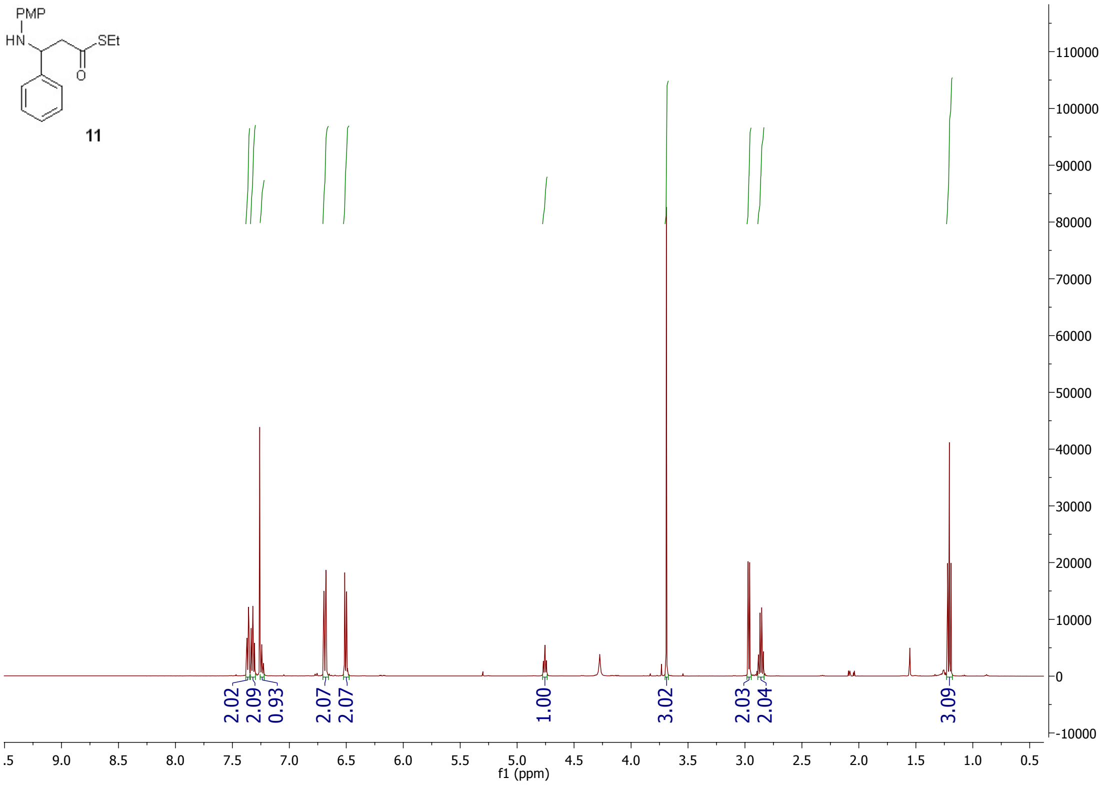
PMP
HN
SEt
O
11
2.02
2.09
0.93
2.07
2.07
1.00
3.02
2.03
2.04
3.09
f1 (ppm)

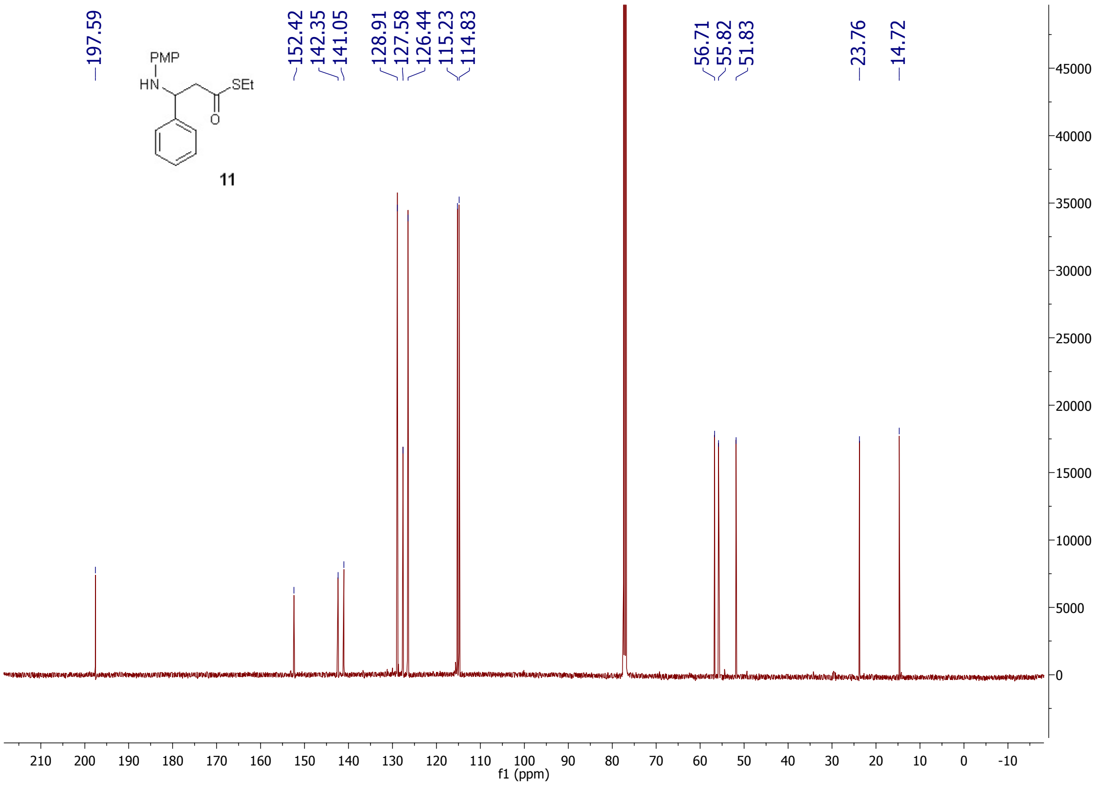
197.59
152.42
142.35
141.05
128.91
127.58
126.44
115.23
114.83
56.71
55.82
51.83
23.76
14.72
PMP
HN
SEt
O
11
f1 (ppm)
